# Inhibition of phosphodiesterase 4D suppresses mTORC1 signaling and pancreatic cancer growth 

**DOI:** 10.1172/jci.insight.158098

**Published:** 2023-07-10

**Authors:** Mi-Hyeon Jeong, Greg Urquhart, Cheryl Lewis, Zhikai Chi, Jenna L. Jewell

**Affiliations:** 1 Department of Molecular Biology,; 2 Harold C. Simmons Comprehensive Cancer Center,; 3 Hamon Center for Regenerative Science and Medicine, and; 4 Department of Pathology, University of Texas Southwestern Medical Center, Dallas, Texas, USA.

**Keywords:** Cell Biology, Cancer

## Abstract

The mammalian target of rapamycin complex 1 (mTORC1) senses multiple upstream stimuli to orchestrate anabolic and catabolic events that regulate cell growth and metabolism. Hyperactivation of mTORC1 signaling is observed in multiple human diseases; thus, pathways that suppress mTORC1 signaling may help to identify new therapeutic targets. Here, we report that phosphodiesterase 4D (PDE4D) promotes pancreatic cancer tumor growth by increasing mTORC1 signaling. GPCRs paired to Gα_s_ proteins activate adenylyl cyclase, which in turn elevates levels of 3′,5′-cyclic adenosine monophosphate (cAMP), whereas PDEs catalyze the hydrolysis of cAMP to 5′-AMP. PDE4D forms a complex with mTORC1 and is required for mTORC1 lysosomal localization and activation. Inhibition of PDE4D and the elevation of cAMP levels block mTORC1 signaling via Raptor phosphorylation. Moreover, pancreatic cancer exhibits an upregulation of PDE4D expression, and high PDE4D levels predict the poor overall survival of patients with pancreatic cancer. Importantly, FDA-approved PDE4 inhibitors repress pancreatic cancer cell tumor growth in vivo by suppressing mTORC1 signaling. Our results identify PDE4D as an important activator of mTORC1 and suggest that targeting PDE4 with FDA-approved inhibitors may be beneficial for the treatment of human diseases with hyperactivated mTORC1 signaling.

## Graphical abstract

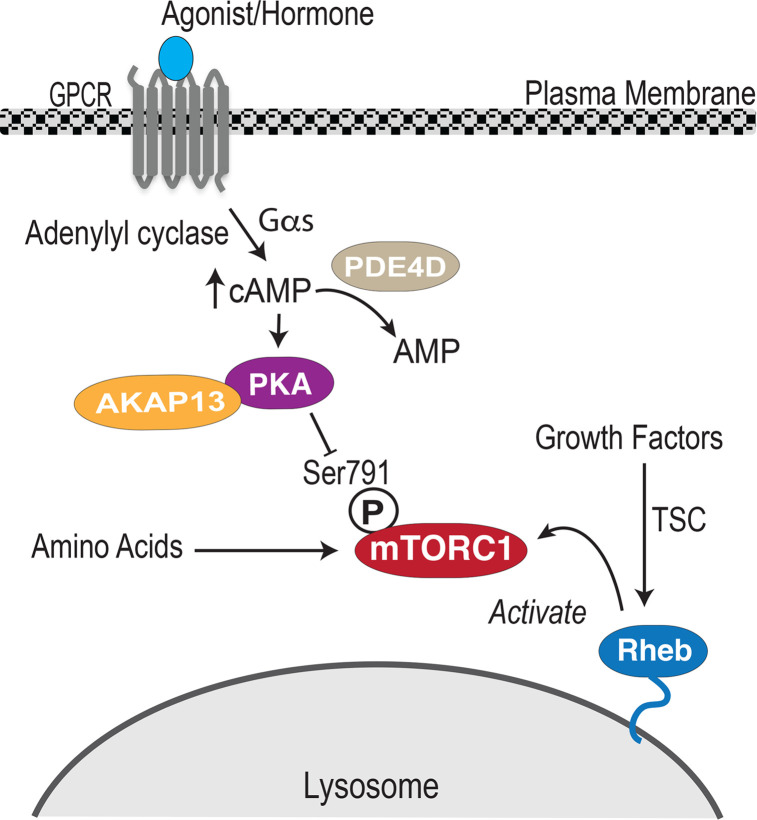

## Introduction

The mammalian target of rapamycin complex 1 (mTORC1) senses multiple upstream stimuli, such as growth factors and amino acids, to regulate cell growth and metabolism (1–4). mTORC1 is composed of the catalytic evolutionarily conserved Ser/Thr protein kinase mTOR, regulatory associated protein of mTOR (Raptor), and mammalian lethal with Sec13 protein 8 (mLST8) (5). Raptor is an mTOR binding partner that recognizes the mTORC1 substrates p70S6 kinase 1 (S6K1) and eIF4E binding protein 1 (4EBP1) (6, 7). mLST8 stabilizes mTOR and promotes mTORC1 signaling (8). Amino acids recruit mTORC1 to the lysosomal surface, resulting in mTORC1 activation (9). Once mTORC1 is activated, it drives protein synthesis through the phosphorylation of S6K1 and 4EBP1, and mTORC1 inhibits autophagy via unc-51-like kinase 1 (ULK1) phosphorylation (3, 10). mTORC1 is abnormally activated in a diverse range of cancers. mTORC1 inhibitors like rapamycin and rapalogs (analogs of rapamycin) are currently used in the clinic, with many limitations (11, 12). Thus, understanding the molecular mechanism involved in mTORC1 regulation, particularly mTORC1 inhibition, is crucial in identifying new potential therapeutic targets.

Ras homolog enriched in brain (Rheb) and the Rag GTPases are crucial for mTORC1 lysosomal translocation and activation (13–15). In response to amino acids, the Rag GTPases recruit mTORC1 to the lysosome where it encounters Rheb. Growth factors signal through Rheb, and GTP-bound Rheb binds to and activates mTORC1 (16, 17). Tuberous sclerosis complex (TSC) is a GTPase-activating protein (GAP) for Rheb, which converts GTP-bound Rheb (active form) to GDP-bound Rheb (inactive form) (18, 19). The Rag GTPases form active heterodimers in which GTP-bound RagA or RagB is in complex with GDP-bound RagC or RagD at the lysosome (20). Amino acids (such as leucine, arginine, and methionine) recruit mTORC1 to the lysosomal surface through the Rag GTPase (21, 22). Other components involved in the Rag GTPase signaling pathway include the Ragulator complex (23, 24), vacuolar H^+^ triphosphate (v-ATPase) (25), the GATOR complex (26), folliculin (27), the KICSTOR complex (28), and solute carrier family 38 member 9 (SLC38A9) (29). We previously discovered a Rag GTPase–independent pathway, where glutamine and asparagine signal to mTORC1 through ADP-ribosylation factor 1 (Arf1) (30, 31). Thus, growth factors and amino acids promote mTORC1 activation at the lysosome.

The second messenger 3′,5′-cyclic adenosine monophosphate (cAMP) is produced by adenylyl cyclase after the activation of specific G protein–coupled receptors (GPCRs) (32). GPCRs are coupled to the G proteins Gα, Gβ, and Gγ. GPCRs coupled to Gα_s_ subunits promote the cAMP signaling pathway. The GTP-bound Gα_s_ subunit dissociates from Gβ and Gγ subunits in response to their corresponding ligands (33, 34). cAMP signaling cascades control a wide range of physiological processes like activating protein kinase A (PKA) (35). PKA is a Ser/Thr kinase consisting of 2 regulatory subunits and 2 catalytic subunits. cAMP binds to the PKA regulatory subunits, resulting in the regulatory subunits dissociating away from the catalytic subunits and PKA activation. PKA phosphorylates a diverse range of substrates, including cAMP response element–binding protein (CREB), which contains the well-characterized PKA phosphorylation motif, Arg-Arg-X-Ser/Thr (RRXS/T) (X represents any amino acid) (36, 37). A-kinase anchoring proteins (AKAPs) bind to regulatory subunits of PKA, localizing the PKA holoenzyme throughout the cell to maintain cell signaling cascades. Increased cAMP levels inhibit cell growth and proliferation through mTORC1 in multiple cancers (38–40). Thus, understanding the crosstalk between cAMP and mTORC1 is important in treating human diseases where mTORC1 is hyperactivated.

Phosphodiesterases (PDEs) are enzymes that degrade cAMP and 3′,5′-cyclic guanosine monophosphate (cGMP) (41, 42). There are 11 families of PDEs that have been identified in mammals. PDE4, PDE7, and PDE8 specifically degrade cAMP. PDE1, PDE2, PDE3, PDE10, and PDE11 hydrolyze both cAMP and cGMP. Inhibitors for PDE4 have been identified as a potential therapeutic strategy to reduce tumor growth and progression (41, 43, 44). PDE4 is encoded by 4 genes, *PDE4A*, *PDE4B*, *PDE4C*, and *PDE4D*, with multiple splice variants in humans (45, 46). Structurally, PDE4 has a conserved catalytic domain in the C-terminus, while the N-terminus contains upstream conserved regions 1 and 2 (UCR1 and UCR2). The UCR1 and UCR2 domains play a role in the dimerization and regulate the catalytic activity of PDE4. Moreover, the UCR1 domain contains a PKA phosphorylation site that increases PDE4 activity for cAMP (47–50). The PDE4 family is divided into 3 subfamilies, depending on the presence or absence of UCR1/2. The PDE4 subfamilies include a long (have UCR1/2), short (have only UCR2), and super-short (have truncated UCR2) form. PDE4D is highly expressed in a variety of cancer types, including pancreatic cancer, lung cancer, breast cancer, and prostate cancer (43, 51–54). Moreover, PDE4D inhibitors (rolipram, roflumilast, and GEBR-7b) have been extensively studied for preventing inflammatory diseases and cancer cell tumor growth specifically in lung cancer and breast cancer (35, 52, 53, 55).

A previous study showed that resveratrol, a polyphenol in red wine, inhibits PDE4 (56). Moreover, resveratrol inhibits mTORC1 signaling. Thus, to better understand the mechanistic details by which mTORC1 is inhibited by GPCRs coupled to Gα_s_, we investigated the role of PDE4D in this pathway. We specifically focused on PDE4D because the depletion of PDE4A, PDE4B, and PDE4C did not alter mTORC1 activity. PDE4D controls cAMP levels, which regulate mTORC1 activity via Raptor Ser791 phosphorylation by PKA. PDE4D interacts with mTORC1 and regulates mTORC1 lysosomal localization. Importantly, the use of US Food and Drug Administration–approved (FDA-approved) PDE4D inhibitors restrains pancreatic cell tumor growth in vivo through mTORC1. Moreover, PDE4D levels play a key role in the overall survival of patients with pancreatic cancer.

## Results

### PDE4D interacts with mTORC1 and regulates its activity.

**Figure 1:**
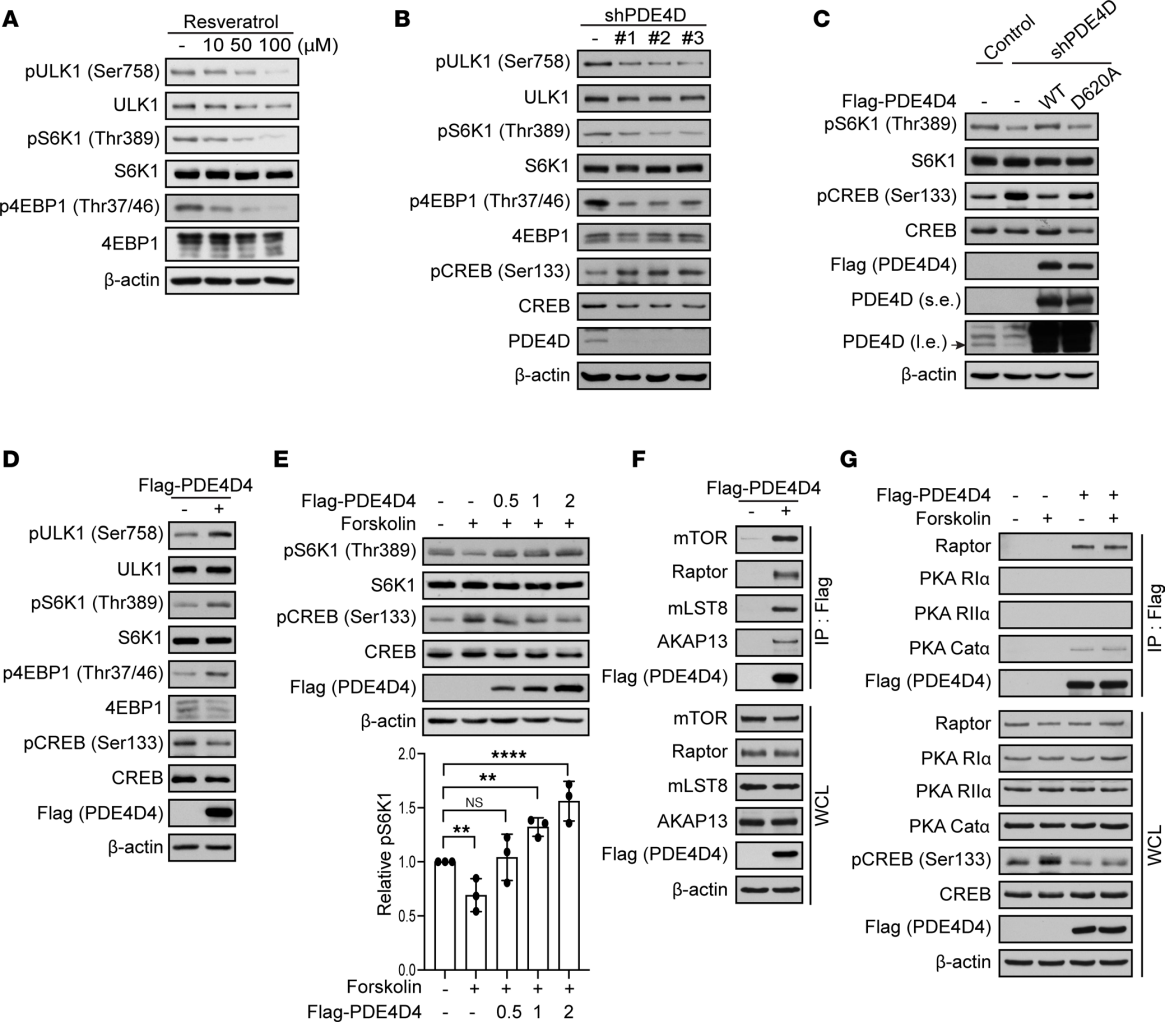
PDE4D forms a complex with mTORC1 and promotes mTORC1 activity. (**A**) Resveratrol inhibits mTORC1 activity. HEK293A cells were treated for 2 hours with resveratrol (0–100 μM). mTORC1 activity was analyzed by immunoblotting for pULK1 (Ser758), pS6K1 (Thr389), and p4EBP1 (Thr37/46). (**B**) Depletion of PDE4D inhibits mTORC1 activity. HEK293A cells were stably generated with 3 shRNAs targeting *PDE4D*. mTORC1 activity was analyzed as in **A**. (**C**) mTORC1 activity is rescued in PDE4D-depleted cells by reintroducing FLAG-tagged PDE4D. Wild-type or an inactive PDE4D mutant (D620A) was expressed in shPDE4D cells. mTORC1 activity was analyzed as in **A**. s.e., short exposure; l.e., long exposure. (**D**) Elevated PDE4D levels increase mTORC1 activity. FLAG-tagged PDE4D4 was overexpressed in cells for 48 hours. mTORC1 activity was analyzed as in **A**. (**E**) Increased PDE4D levels promote mTORC1 activity. FLAG-tagged PDE4D4 (0.5–2 μg) was transfected into HEK293A cells for 48 hours, and then stimulated with forskolin (10 μM) for 1 hour. mTORC1 activity was analyzed as in **A**. The quantification of pS6K1 was analyzed using ImageJ and normalized to S6K1. The data represent mean ± SD of triplicate experiments. NS, not significant. **P < 0.01, ****P < 0.0001 by 1-way ANOVA with Dunnett’s test for multiple comparisons. (**F**) PDE4D binds to mTORC1 and AKAP13. Cells were transfected with FLAG-tagged PDE4D4 for 48 hours and then lysates immunoprecipitated (IP) with anti-FLAG antibody (PDE4D4). mTORC1 (mTOR, Raptor, mLST8), AKAP13, and FLAG (PDE4D4) were probed for. WCL, whole-cell lysates. (**G**) PDE4D interacts with PKA catalytic subunits. FLAG-tagged PDE4D4 was overexpressed in cells and cells were treated with forskolin (10 μM) for 1 hour. Lysates were immunoprecipitated with anti-FLAG antibody (PDE4D4). Raptor, PKA RIα, PKA RIIα, PKA Catα, pCREB (Ser133), CREB, FLAG (PDE4D4), and β-actin were probed for. Immunoblots probed for ULK1, S6K1, 4EBP1, β-actin, pCREB (PKA activation), CREB, PDE4D, and FLAG are controls.

Increased GPCR/Gα_s_ signaling elevates cAMP levels, resulting in the potent inhibition of mTORC1 (57). Since PDEs regulate cAMP levels and there are many FDA-approved PDE inhibitors (45, 58, 59), we have been actively looking for the PDE involved in this pathway. A polyphenol in red wine called resveratrol was previously shown to inhibit PDE4 (56), and resveratrol inhibits mTORC1 in a dose-dependent manner in HEK293A cells (Figure 1A). mTORC1 activity was analyzed by the phosphorylation of its substrates (S6K1 at Thr389, 4EBP1 at Thr37 and Thr46, and ULK1 at Ser758). S6K1 and 4EBP1 phosphorylation promote protein synthesis and ULK1 phosphorylation inhibits autophagy (5, 60). PDE4 is encoded by 4 distinct genes, *PDE4A*–*D*, with more than 20 different isoforms in mammals (61). Interestingly, the depletion of PDE4D significantly decreased mTORC1 activity (Figure 1B), and the overexpression of FLAG-tagged PDE4D4 rescued PDE4D-depleted mTORC1 inhibition (Figure 1C). In addition, a catalytically inactive mutant, PDE4D4 D620A (Asp620 mutated to Ala620; the Asp620 residue is conserved in all PDE4 isoforms, refs. 62, 63), fails to rescue *PDE4D*-knockdown–mediated inhibition of mTORC1. In contrast to PDE4D, the depletion of PDE4A–C did not alter the activation of mTORC1 (Supplemental Figure 1; supplemental material available online with this article; https://doi.org/10.1172/jci.insight.158098DS1). Consistently, overexpression of FLAG-tagged PDE4D4 or FLAG-tagged PDE4D6 increased mTORC1 activity (Figure 1D and Supplemental Figure 2A). PDE4D4 (809 amino acids) is the longest PDE4D isoform containing the UCR1/UCR2 and catalytic domains, whereas PDE4D6 (518 amino acids) is a shorter isoform that contains a truncated UCR2 and catalytic domain (Supplemental Figure 2B). We previously found that GPCRs paired to Gα_s_ proteins increase cAMP levels to inhibit the activity of mTORC1 (57). Specifically, cAMP levels activate PKA to phosphorylate Raptor on Ser791, resulting in mTORC1 inhibition. As expected, cells treated with forskolin, a pharmacological activator of adenyl cyclase, resulted in decreased mTORC1 activity (Figure 1E). Overexpression of FLAG-tagged PDE4D4 relieved cAMP-induced mTORC1 inhibition. Moreover, FLAG-tagged PDE4D6 and PDE4D4 formed a complex with mTORC1 and PKA catalytic subunit α (PKA Catα) (Figure 1, F and G, and Supplemental Figure 2, C–E). Endogenous mTORC1 also interacted with PDE4D (Supplemental Figure 2E). We recently found out that A-kinase anchoring protein 13 (AKAP13) regulates mTORC1 activity by scaffolding PKA near mTORC1 (64). Interestingly, AKAP13 only associated with PDE4D4, containing the UCR1 domain at the N-terminus and not PDE4D6 (Figure 1F and Supplemental Figure 2D). Thus, PDE4D interacts with and regulates mTORC1 activity.

### PDE4D regulates mTORC1 activity through Raptor Ser791 phosphorylation.

**Figure 2:**
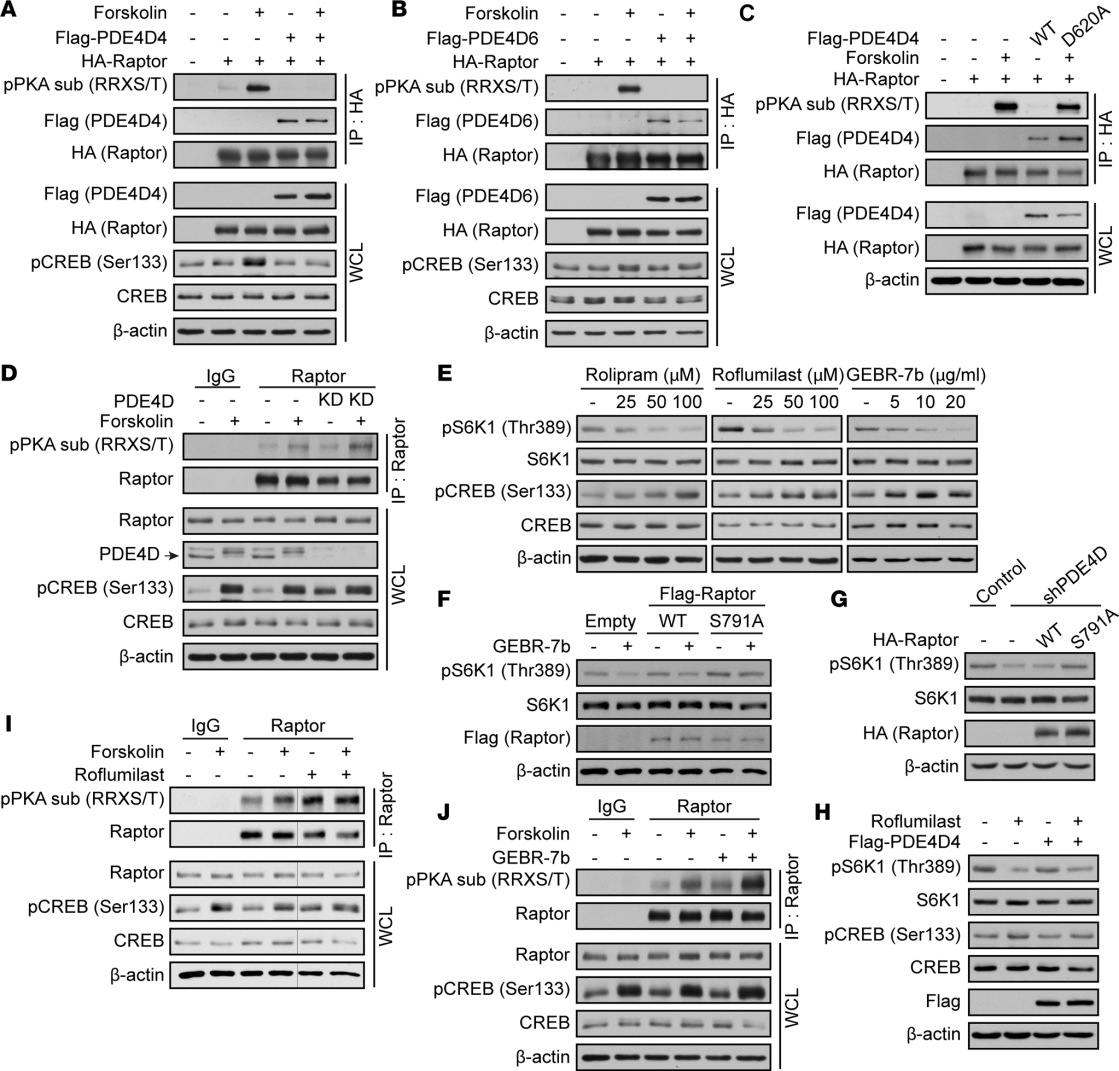
PDE4D promotes mTORC1 activity through Raptor Ser791 phosphorylation. (**A** and **B**) PDE4D inhibits Raptor Ser791 phosphorylation. (**A**) HA-tagged Raptor was coexpressed with FLAG-tagged PDE4D4 in HEK293A cells for 48 hours; cells were then treated with forskolin (10 μM) for 1 hour. Lysates were immunoprecipitated (IP) with anti-HA antibody and immunoblotted with anti-pPKA substrate (RRXS/T) antibody. (**B**) Same as **A** but with FLAG-tagged PDE4D6. (**C**) PDE4D controls Raptor Ser791 phosphorylation. FLAG-tagged PDE4D (WT), FLAG-tagged PDE4D catalytically inactive mutant (D620A), and HA-tagged Raptor were overexpressed. Cells were treated with forskolin (10 μM) for 1 hour. Lysates were immunoprecipitated with anti-HA antibody and immunoblotted with anti-pPKA substrate antibody. (**D**) PDE4D depletion enhances Raptor Ser791 phosphorylation. Cells with PDE4D shRNA were stimulated with forskolin (10 μM) for 1 hour. Lysates were immunoprecipitated with anti-Raptor antibody and immunoblotted for pPKA substrate. Arrow indicates PDE4D. (**E**) Chemical inhibition of PDE4D decreases mTORC1 activity. HEK293A cells were stimulated with rolipram, roflumilast, and GEBR-7b for 2 hours and mTORC1 activity was analyzed. (**F** and **G**) Raptor Ser791 phosphorylation controls mTORC1 activity. (**F**) HEK293A cells expressing FLAG-tagged Raptor (WT) or FLAG-tagged Raptor S791A (phospho-defective) were treated with or without GEBR-7b (20 μg/mL) for 2 hours. mTORC1 activity was analyzed. (**G**) HA-tagged Raptor (WT) or HA-tagged Raptor S791A (phospho-defective) was overexpressed in PDE4D-depleted cells (shPDE4D). mTORC1 activity was analyzed. (**H**) PDE4D controls mTORC1 activity. Cells were transfected with FLAG-tagged PDE4D4 for 48 hours and stimulated with roflumilast (50 μM) for 2 hours. mTORC1 activity was analyzed. (**I** and **J**) Pharmacologic inhibition of PDE4D enhances Raptor Ser791 phosphorylation. HEK293A cells were pretreated with either roflumilast (50 μM) (**I**) or GEBR-7b (20 μg/m) (**J**) for 1 hour and then treated with forskolin (10 μM) for 1 hour. Lysates were immunoprecipitated with anti-Raptor antibody and Raptor Ser791 phosphorylation was assessed. Immunoblots probed for Raptor, pCREB, CREB, FLAG, HA, S6K1, and β-actin are controls. WCL, whole-cell lysates.

PKA is a cAMP-dependent protein kinase and phosphorylates Ser/Thr residues on substrates, preferably on the Arg-Arg-X-Ser/Thr (RRXS/T, where X tends to be a hydrophobic residue and Ser/Thr are the phosphorylatable residues) recognition motif (65). Raptor Ser791 is the only Ser/Thr residue that resides within the recognition motif. We previously demonstrated through site-directed mutagenesis and in vitro kinase assays that PKA directly phosphorylates Raptor at Ser791 (57). Because PDE4D controls cAMP levels and regulates mTORC1 activity (Figure 1, B–E, and Supplemental Figure 2A), we investigated whether PDE4D inhibited mTORC1 through Raptor Ser791 phosphorylation. HA-tagged Raptor was coexpressed with either FLAG-tagged PDE4D4 or FLAG-tagged PDE4D6 in HEK293A cells that were treated with or without forskolin (Figure 2, A and B). HA-tagged Raptor was immunoprecipitated and Raptor on Ser791 phosphorylation was analyzed using a phospho-PKA substrate antibody (pPKA sub [RRXS/T]). This antibody recognizes Raptor when it becomes phosphorylated on Ser791 (57). The expression of FLAG-tagged PDE4D4 or FLAG-tagged PDE4D6 decreased Raptor Ser791 phosphorylation, consistent with elevated levels of PDE4D activating mTORC1 (Figure 1D and Supplemental Figure 2A). However, overexpression of FLAG-tagged PDE4D4 D620A failed to decrease Raptor phosphorylation at Ser791 (Figure 2C). In contrast, the depletion of PDE4D enhanced Raptor Ser791 phosphorylation after forskolin treatment (Figure 2D). Because the endogenous PDE4D band runs more slowly (shifted up) in response to forskolin treatment (Figure 2D) and the UCR1 domain of PDE4 contains a PKA phosphorylation site (47–50), we investigated whether PDE4D phosphorylation by PKA altered Raptor Ser791 phosphorylation. PDE4D has 2 predicted PKA substrate motifs, RRXS/T on Ser190 and KKXS/T (36, 66, 67) on Thr595. There was an increase in PDE4D phosphorylation in response to forskolin treatment as determined by phospho-PKA substrate antibody (Supplemental Figure 3A). A phospho-defective Ser190 (Ser190 to Ala190, S190A) mutation on PDE4D was resistant to RRXS/T phosphorylation in response to forskolin treatment (Supplemental Figure 3B). In addition, we made a phospho-defective Thr595 (Thr595 to Ala595, T595A) mutation on PDE4D4. Overexpression of FLAG-tagged PDE4D4 was still able to decrease Raptor Ser791 phosphorylation even when FLAG-tagged PDE4D was mutated (S190A and T595A) (Supplemental Figure 3C). The phosphorylation of PDE4D4 by PKA does not alter Raptor Ser791. Taken together, these results show that PDE4D regulates mTORC1 activity through Raptor Ser791 phosphorylation.

Rolipram (68) and roflumilast (69) are PDE4 inhibitors and approved by the FDA. Moreover, GEBR-7b has been shown to be a selective inhibitor for PDE4D (70). Importantly, rolipram, roflumilast, and GEBR-7b inhibited mTORC1 in a dose-dependent manner (Figure 2E). A phospho-defective Ser791 (Ser791 to Ala791, S791A) on Raptor relieved mTORC1 inhibition by the PDE4D inhibitor (GEBR-7b) (Figure 2F) or PDE4D knockdown (Figure 2G), showing that PDE4D controls mTORC1 signaling through Raptor Ser791 phosphorylation. Moreover, overexpression of FLAG-tagged PDE4D4 rescued roflumilast-mediated mTORC1 inhibition (Figure 2H). Likewise, treatment of cells with roflumilast or GEBR-7b significantly enhanced Raptor Ser791 phosphorylation (Figure 2, I and J). Thus, the FDA-approved inhibitors block mTORC1 activity via Raptor Ser791 phosphorylation. The use of PDE4 inhibitors to treat diseases with hyperactivated mTORC1 may be useful.

### PDE4D regulates mTORC1 trafficking to the lysosome.

**Figure 3:**
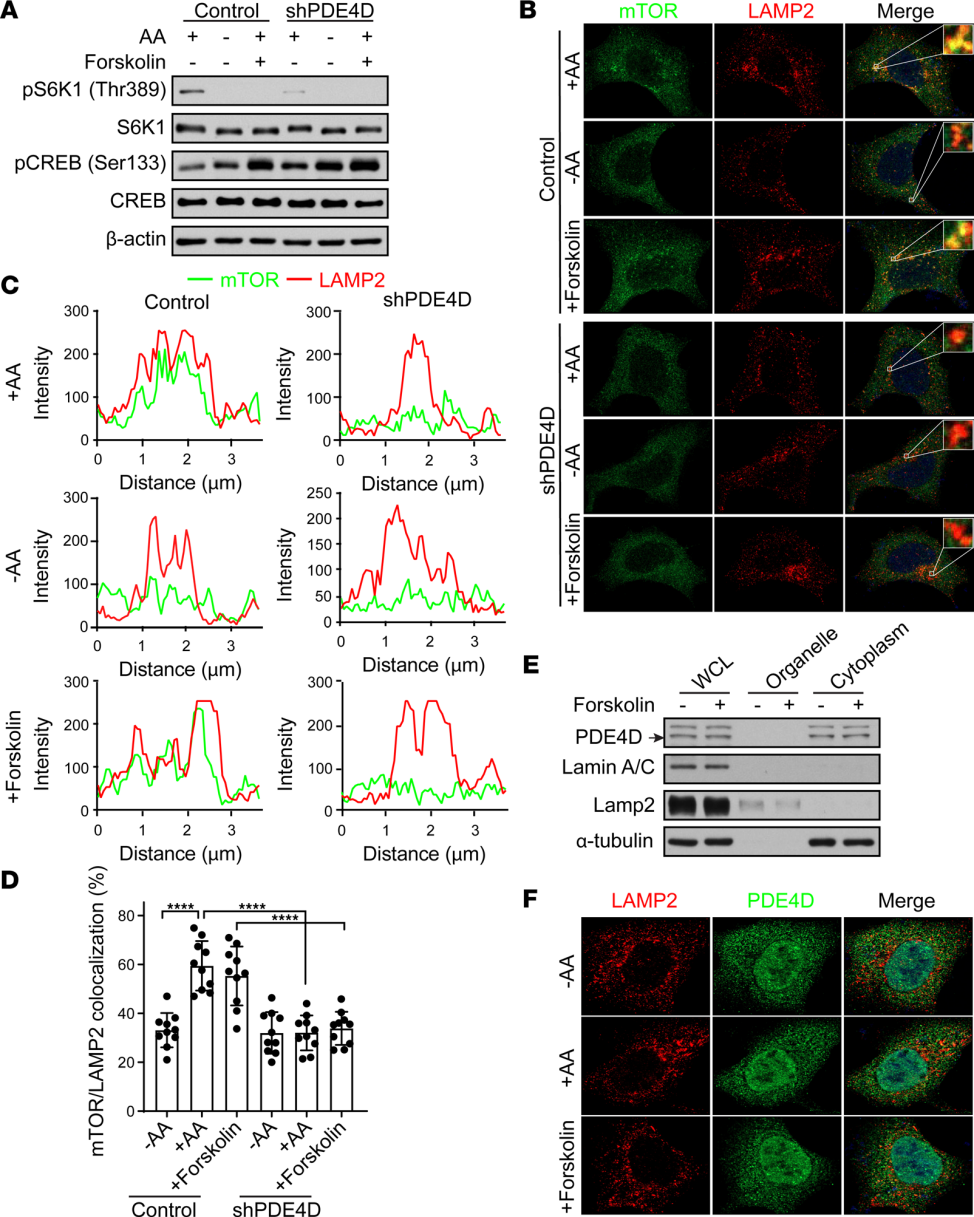
PDE4D promotes mTORC1 lysosomal localization. (**A**) Depletion of PDE4D inhibits amino acid–induced mTORC1 activation. HEK293A cells stably expressing shGFP (control) or shRNA targeting *PDE4D* (shPDE4D) were generated. Cells were starved in amino acid–free media for 2 hours, pretreated with or without forskolin (10 μM) for 1 hour, and then stimulated with amino acids for 1 hour. mTORC1 activity with pS6K1 (Thr389) was analyzed. S6K1, pCREB (Ser133) (measure of PKA activation), CREB, and β-actin are controls. (**B**–**D**) Depletion of PDE4D blocks mTORC1 lysosomal localization. (**B**) HEK293A cell lines stably expressing shGFP (control) or shPDE4D were starved in amino acid–free media for 2 hours, pretreated with or without forskolin (10 μM) for 1 hour, and then stimulated with amino acids for 1 hour. Immunofluorescence experiments were performed with anti-mTOR (green) and -LAMP2 (lysosome marker, red) antibodies. Representative images were obtained under a Zeiss LSM 900 confocal microscope with 100× objective. (**C**) Staining intensity profiles across the 3.6-μm distance of the green (mTOR) and red (LAMP2) channels in the magnified pictures. (**D**) Ten immunofluorescence images per group were quantified using Squassh in ImageJ. The data represent mean ± SD. ****P < 0.0001 by 2-way ANOVA with Tukey’s test for multiple comparisons. (**E**) PDE4D localizes in the cytoplasm. Cells were fractionated after forskolin (10 μM) treatment for 1 hour and then analyzed by immunoblotting for PDE4D. LAMP2 (lysosome marker), lamin A/C (nuclear marker), and α-tubulin (cytoplasm marker) are controls. Arrow indicates PDE4D. (**F**) PDE4D does not localize to the lysosome. HEK293A were starved in amino acid–free media for 2 hours, pretreated with or without forskolin (10 μM) for 1 hour, and then stimulated with amino acids for 1 hour. Immunofluorescence experiments were performed with anti-PDE4D (green) and -LAMP2 (lysosome marker, red) antibodies. Representative images were obtained under an LSM 900 confocal microscope with 100× objective. AA, amino acid.

mTORC1 translocates to the lysosomal surface in response to amino acids, where it is activated by the small GTPase Rheb (71). mTORC1 is not at the lysosome when cells are depleted of amino acids and is thought to be dispersed throughout the cell at an unknown location (9). We previously reported that increased intracellular cAMP levels can inhibit amino acid signaling to mTORC1 (57). However, altering intracellular cAMP levels did not impact mTORC1 lysosomal localization, but still blocked amino acid–induced mTORC1 activation through an unknown mechanism. Because PDE4D interacts with and regulates mTORC1 activity (Figure 1, B–G, and Supplemental Figure 2, C–E) and PDE4D depletion inhibits amino acid–induced mTORC1 activation (Figure 3A), we investigated whether PDE4D played a role in mTORC1 lysosomal localization (Figure 3, B–D, and Supplemental Figure 4, A and B). As expected, amino acids promoted mTORC1 lysosomal localization in control cells, whereas mTORC1 was not present at the lysosome under amino acid starvation conditions. And as previously seen, increasing intracellular cAMP levels with forskolin did not alter mTORC1 lysosomal localization in response to amino acids. Interestingly, the depletion of PDE4D by knockdown or pharmacological inhibition significantly impaired mTORC1 lysosomal localization in response to amino acid stimulation with or without forskolin treatment. This result suggests that PDE4D somehow plays a role in mTORC1 lysosomal localization, possibly through the PDE4D-mTORC1 interaction. It has been reported that PDE4D is localized to cytosolic compartments in mouse embryonic fibroblasts (72). Consistent with this, PDE4D localized in the cytoplasm in HEK293A cells (Supplemental Figure 4C), and subcellular fractionation experiments revealed that PDE4D appeared to reside in the cytoplasm (not at the lysosome) regardless of cAMP stimulation (Figure 3, E and F). Taken together, these results show that PDE4D promotes mTORC1 recruitment to lysosomes in response to amino acids by a mechanism independent of cAMP levels.

### Pharmacologic PDE4D inhibition suppresses mTORC1 signaling and pancreatic cancer growth in vivo.

**Figure 4:**
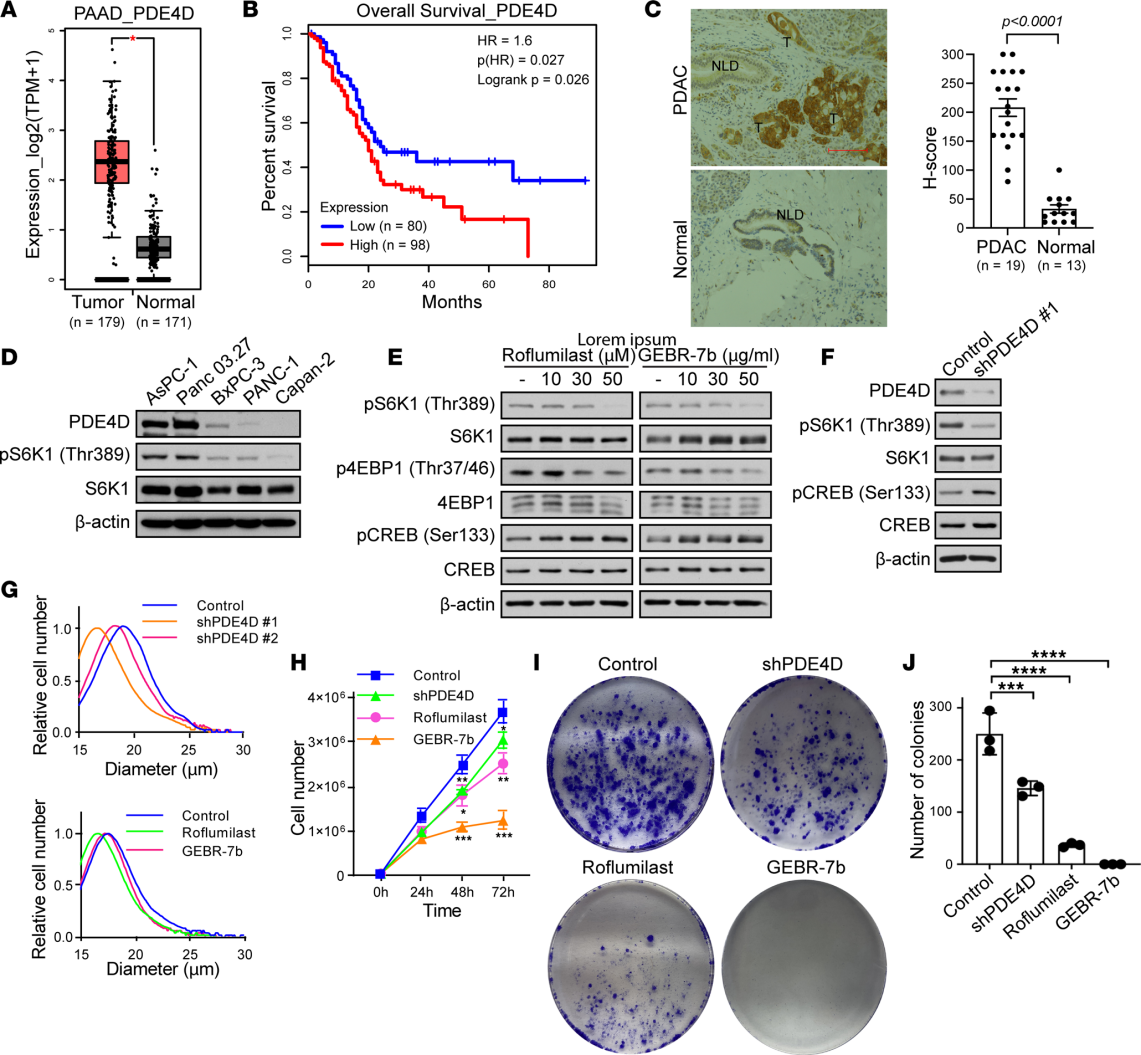
Pharmacologic PDE4D inhibition impairs mTORC1 signaling and pancreatic cancer cell growth. (**A**) High PDE4D levels in patients with pancreatic adenocarcinoma from GEPIA (74). *P < 0.05. (**B**) High PDE4D has low survival rate in patients with pancreatic adenocarcinoma (74). Log-rank test was performed for statistical analysis (**A** and **B**). (**C**) High PDE4D expression in patients with pancreatic ductal adenocarcinoma. Pancreatic ductal adenocarcinoma (PDAC) tissue sections paired with benign tissues. T, tumor; NLD, normal duct. Scale bar: 100 μm. Significance was assessed by 2-tailed Student’s *t* test. (**D**) PDE4D levels correlate with mTORC1 activity in pancreatic cancer cells. (**E** and **F**) PDE4D inhibition blocks mTORC1 activity. (**E**) MIA PaCa-2 cells were treated with roflumilast or GEBR-7b for 2 hours. mTORC1 activity was analyzed. (**F**) MIA PaCa-2 cells stably expressing shPDE4D have low mTORC1 activity. (**G**) PDE4D inhibition reduces pancreatic cancer cell size. Cell size was determined for shPDE4D MIA PaCa-2 cells, or MIA PaCa-2 cells treated with roflumilast or GEBR-7b for 48 hours. One-way ANOVA with Dunnett’s test was performed. *P* < 0.0001 for control vs. shPDE4D #1. *P* < 0.05 for control vs. shPDE4D #2 and control vs. GEBR-7b. *P* < 0.001 for control vs. roflumilast. (**H**) Pharmacological inhibition of PDE4D reduces proliferation of pancreatic cancer cells. MIA PaCa-2 cells with shPDE4D, or MIA PaCa-2 cells treated with roflumilast or GEBR-7b were counted. Data represent mean ± SD. *P < 0.05, **P < 0.01, ***P < 0.001 by 2-way ANOVA with Dunnett’s multiple-comparison test. (**I** and **J**) Pharmacological inhibition of PDE4D reduces colony formation. (**I**) Colony formation assays in MIA PaCa-2 cells 2 weeks after seeding cells (1 × 10^3^). (**J**) Colonies in **I** were counted using ImageJ for the quantification. Bar graph represents mean ± SD. ***P < 0.001, ****P < 0.0001 by 1-way ANOVA with Dunnett’s multiple-comparison test. Immunoblots probed for PDE4D, S6K1, 4EBP1, pCREB (Ser133) (measure of PKA activation), CREB, and β-actin are controls.

mTORC1 modulates fundamental physiological processes like cell growth, and dysregulation of mTORC1 signaling can result in human cancer (5, 10, 73). Analysis of The Cancer Genome Atlas (TCGA) data indicates that PDE4D expression is high in patients with pancreatic adenocarcinoma (Figure 4A). Moreover, data obtained from gene expression profiling interactive analysis (GEPIA) show that high PDE4D expression is associated with poor survival of patients with pancreatic adenocarcinoma (Figure 4B) (74). We confirmed that PDE4D expression is higher in tissues of patients with pancreatic ductal adenocarcinoma (PDAC) compared with paired benign tissues (Figure 4C and Supplemental Table 1). Moreover, the level of PDE4D correlates with mTORC1 activity in pancreatic cancer cells (Figure 4D). Like HEK293A cells (Figure 2E), the PDE4 inhibitors roflumilast and GEBR-7b inhibited mTORC1 in pancreatic cancer MIA PaCa-2 cells (Figure 4E). Moreover, depletion of PDE4D inhibited mTORC1 signaling in MIA PaCa-2 cells (Figure 4F). MIA PaCa-2 size and proliferation were significantly reduced, similar to that of mTORC1 signaling, when PDE4D was either depleted or pharmacologically inhibited (roflumilast or GEBR-7b) (Figure 4, G and H, and Supplemental Figure 5). Put-back experiments overexpressing FLAG-tagged PDE4D4 rescued mTORC1 activity and cell growth (cell size and proliferation) when PDE4D was depleted, whereas inactive FLAG-tagged PDE4D4 D620A was unable to rescue (Supplemental Figure 6, A–C). A phospho-defective S791 (Ser791 mutated to Ala791) in Raptor relieved mTORC1 inhibition and decreased cell growth (cell size and proliferation) in PDE4D-depleted MIA PaCa-2 cells (Supplemental Figure 7, A–C). PDE4D depletion or inhibition also inhibited colony formation (Figure 4, I and J). Interestingly, it appears that PDE4D regulates mTORC1 activity, cell size, and cell proliferation in KRAS-mutant (AsPC-1) compared with non–KRAS-mutant (BxPC-3) pancreatic cancer cells (Supplemental Figure 8, A–C). Collectively, these results demonstrate that PDE4D inhibition prevents pancreatic cell growth, cell proliferation, and colony formation.

**Figure 5:**
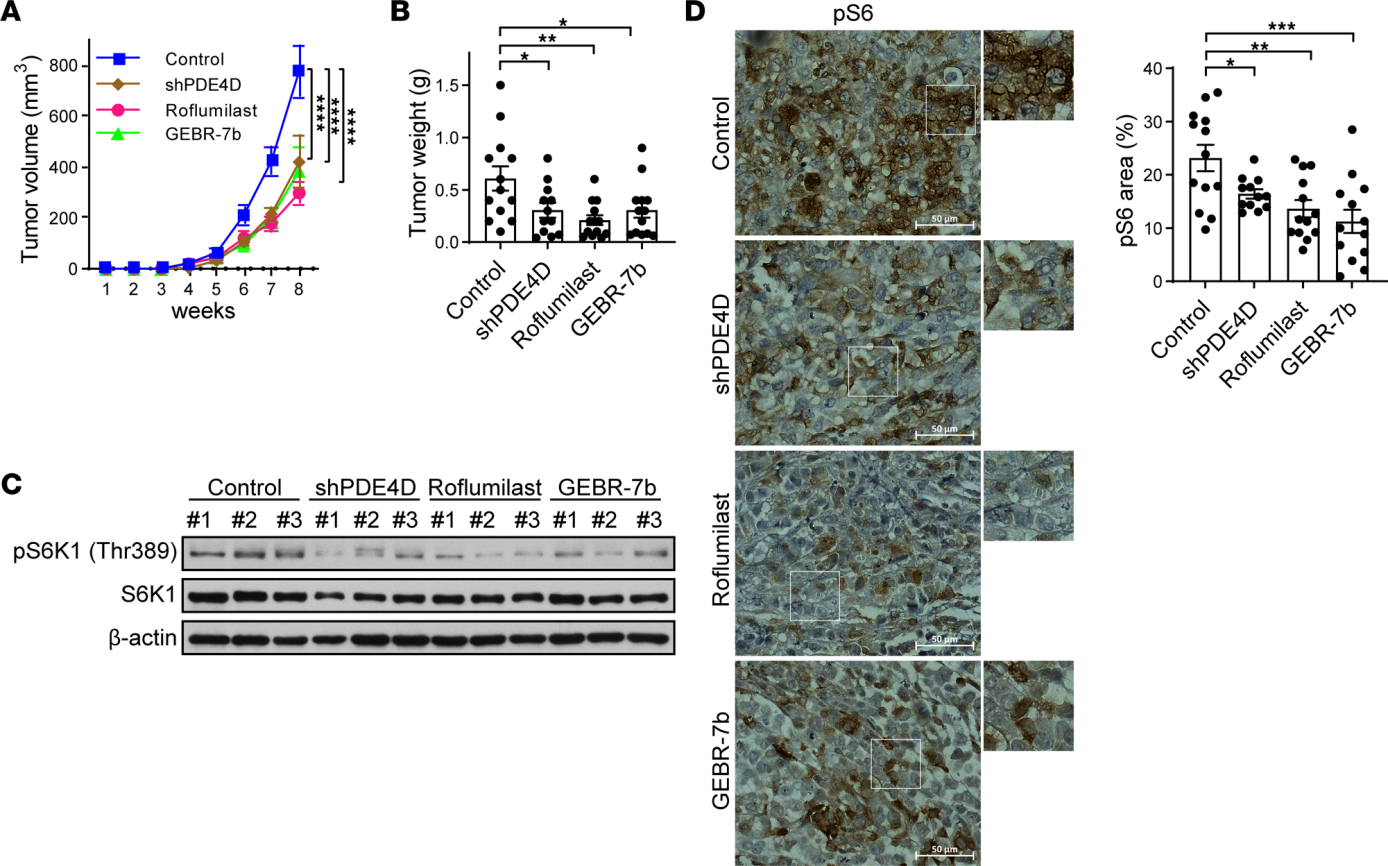
Targeting PDE4D suppresses in vivo tumor development of pancreatic cancer. (**A** and **B**) PDE4D inhibition suppresses tumor growth. MIA PaCa-2 cells stably expressing shGFP (control) or shPDE4D were subcutaneously injected into mice for tumor xenograft experiments. In addition, MIA PaCa-2 cells were subcutaneously injected into mice for tumor xenograft experiments and injected with either roflumilast (5 mg/kg) or GEBR-7b (0.3 mg/kg) intraperitoneally once every other day. Tumor growth in **A** was measured every week after injecting cancer cells into mice. Tumor weight in **B** was measured after resection of xenograft tumor from mice. Control *n* = 13, shPDE4D *n* = 12, roflumilast *n* = 13, and GEBR-7b *n* = 13. (**C** and **D**) PDE4D inhibition suppresses mTORC1 activity in pancreatic cancer. The xenograft tumor samples were assessed by immunoblotting (**C**) for mTORC1 activity via pS6K1. S6K1 and β-actin are controls. The xenograft tumor samples were assessed by immunohistochemistry (**D**) for pS6 (Ser235/236). Quantification of immunohistochemical staining was obtained using ImageJ. Scale bars: 50 μm. Data represent mean ± SEM. *P < 0.05, **P < 0.01, ***P < 0.001, ****P < 0.0001 by 2-way ANOVA with Dunnett’s multiple-comparison test (**A**) or 1-way ANOVA with Dunnett’s multiple-comparison test (**B** and **C**).

Next we investigated the role of PDE4D in vivo. MIA PaCa-2 control or PDE4D-depleted cells were injected subcutaneously into mice. Tumor volume and weight were significantly decreased in PDE4D-depleted cells. Consistently, tumor volume and weight were lower in mice injected with PDE4D inhibitors (Figure 5, A and B). Tumors isolated from mice showed a reduction in mTORC1 signaling when PDE4D was depleted or inhibited (Figure 5C). Immunohistochemical experiments also revealed a decrease in mTORC1 activity when PDE4D was knocked down or inhibited using an anti-pS6 antibody (measure of mTORC1 activity) (Figure 5D). This anti-pS6 antibody has been validated in mice using rapamycin and other inhibitors upstream of mTORC1 (75–77). Taken together, these results show that the pharmacological inhibition of PDE4D results in a decreased mTORC1 signaling and tumor growth in vivo.

## Discussion

The GPCR/Gα_s_ signaling pathway decreases cell growth in a variety of tumor types, including skin cancer and medulloblastoma (78–80). PDEs degrade cAMP to negatively modulate GPCR/Gα_s_ signaling (32, 45). We previously reported that elevated intracellular cAMP levels inhibit mTORC1 by activating PKA to phosphorylate Raptor at Ser791 (57). Here, we show that PDE4D decreases Raptor Ser791 phosphorylation and promotes mTORC1 signaling. In contrast to PDE4D, other PDE4 family members (PDE4A, PDE4B, and PDE4C) did not alter mTORC1 activity in our studies. However, a previous study reported that PDE4A is involved in autophagy-related 5– and 7– (ATG5- and ATG7-dependent) autophagy induced by yessotoxin, a potential antiallergic and anticancer drug mediated by mTOR in erythroleukemia cells (81). Similarly, PDE4B was shown to modulate colorectal cancer growth through mTOR (82). Thus, it is possible that different PDE4 family members may play a role in cell proliferation and tumor development through mTORC1 in different types of cancers. Among the PDE family members, PDE4, PDE7, and PDE8 hydrolyze cAMP (41, 42). PDE1, PDE2, PDE3, PDE10, and PDE11 can hydrolyze cAMP and cGMP. The potential role of additional PDEs that hydrolyze cAMP in mTORC1 regulation remains to be investigated.

In response to amino acids, mTORC1 is localized to the lysosome and activated via a Rag GTPase–dependent (23) or Rag GTPase–independent (31) pathway. We discovered that PDE4D is required for mTORC1 recruitment to the lysosome in the presence of amino acids. Because the elevation of cAMP by forskolin does not alter mTORC1 lysosomal localization, it is possible that PDE4D plays a role in mTORC1 lysosomal localization via the PDE4D-mTORC1 interaction. Perhaps PDE4D promotes the trafficking of mTORC1 to the lysosome. For example, PDE4D is predicted to interact with the lysosome trafficking protein Abelson murine leukemia viral oncogene homolog 1 (ABL1) by BioGRID (83), suggesting that PDE4D may have a role in the compartmentalization of mTORC1 throughout the cell.

GPCRs constitute the largest category of FDA-approved drugs on the market today (84). Similarly, PDE4 inhibitors, including roflumilast, cilomilast, and rolipram, have been approved by the US FDA for treatment of inflammation and chronic obstructive pulmonary disease (55). We believe that targeting GPCR/Gα_s_ signaling in combination with using PDE inhibitors may be beneficial in human diseases with hyperactivated mTORC1.

## Methods

### Cell culture and reagents.

HEK293A, PANC-1, and MIA PaCa-2 cell lines (including PDE4D-knockdown cells) were maintained in high-glucose DMEM (Invitrogen) supplemented with 10% FBS (Sigma-Aldrich) and 1% penicillin/streptomycin (Sigma-Aldrich) at 37°C with 5% CO_2_. Panc 03.27 cells were cultured in RPMI-1640 basal medium supplemented with 15% FBS, 10 U/mL human recombinant insulin, and 1% penicillin/streptomycin. AsPC-1, BxPC-3, and Capan-2 cell lines were maintained in RPMI-1640 supplemented with 10% FBS and 1% penicillin/streptomycin. Cells were transfected with PolyJet in vitro DNA transfection reagent (Thermo Fisher Scientific) following the manufacturer’s protocol. Forskolin was purchased from Tocris. Resveratrol, rolipram, roflumilast, and GEBR-7b were purchased from Sigma-Aldrich.

### Plasmids.

Relevant PDE4D (PDE4D4 and PDE4D6) constructs encoding wild-type, S190A, S190A/T595A, and D620A of PDE4D4 were generated by PCR and cloned into FLAG-pcDNA3.1 and lentiCRISPR v2 with a FLAG tag. The generation of FLAG- or HA-tagged Raptor wild-type and S791A constructs was described in previous work (57).

### Generation of stable cell lines.

shRNA plasmid DNA against *PDE4D* (1: TRCN0000236065, 2: TRCN000048837, 3: TRCN000048835) were purchased from Sigma-Aldrich. FLAG-tagged or HA-tagged Raptor wild-type and S791A constructs were cloned into lentiCRSPR v2. To generate lentiviral particles, packaging plasmids pMD2.G and psPAX2 (Addgene) were cotransfected with pLKO.1-PDE4D and lentiCRSPR v2-Raptor constructs into HEK293A cells. The supernatant was collected and centrifuged at 3000*g* for 10 minutes after 48 hours. HEK293A, MIA PaCa-2, AsPC-1, and BxPC-3 cells were infected with lentiviral particles using 8 μg/mL polybrene (Sigma-Aldrich), and then selected with 2 μg/mL puromycin (Sigma-Aldrich) and 10 μg/mL blasticidin.

### siRNA transfection.

Cells were transiently transfected using DharmaFECT transfection reagent (Dharmacon) according to the manufacturer’s procedure. ON-TARGET plus SMART pool siRNAs (Dharmacon) against *PDE4A* (L-007647-00-0005), *PDE4B* (L-007648-01-0005), *PDE4C* (L-007649-02-0005), and *PDE4D* (L-004757-02-0005) were used at 50 nM.

### Immunoprecipitation and immunoblot analysis.

Cells were lysed with CHAPS buffer (40 mM HEPES pH 7.5, 120 mM NaCl, 1 mM EDTA, 10 mM sodium pyrophosphate, 10 mM glycerol-2-phosphate, 50 mM NaF, 0.5 mM sodium orthovanadate, Complete EDTA-free protease tablet [Roche], and 0.3% CHAPS). The lysates were briefly vortexed and cleared by centrifugation at 13,000 rpm for 10 minutes at 4°C. The cleared supernatants were transferred to fresh tubes and were ready to use for immunoprecipitation with anti-HA magnetic beads (Thermo Fisher Scientific), anti-FLAG-M2 agarose beads (Sigma-Aldrich), and Protein A/G magnetic beads (Thermo Fisher Scientific). Equal quantities of protein extracts and immunoprecipitation products were electrophoresed in SDS-polyacrylamide gels and then transferred to polyvinylidene difluoride membranes (Bio-Rad). The membranes were blocked with 5% (w/v) skim milk in Tris buffer pH 7.4 containing 0.1% (v/v) Tween 20 (Sigma-Aldrich) for 1 hour and then were probed with primary antibodies and HRP-conjugated secondary antibodies. Antibodies against the following proteins were used: β-actin (catalog 3700), HA tag (catalog 3724), pULK1 (catalog 6888), ULK1 (catalog 8054), pS6K1 (catalog 9234), S6K1 (catalog 9202), p4EBP1 (catalog 2855), 4EBP1 (catalog 9452), pCREB (catalog 9198), CREB (catalog 9197), mTOR (catalog 2972), mLST8 (catalog 3274), PKA Catα (catalog 4782), PKA RIα (catalog 5675), pPKA substrate (RRXS/T; catalog 9621) (all Cell Signaling Technology); Raptor (Cell Signaling Technology, catalog 2280 and Bethyl Laboratories, catalog A300-553A), AKAP13 (Thermo Fisher Scientific, catalog PA554078), PKA RIIα (Bethyl Laboratories, catalog A301-670A-M), FLAG tag (Sigma-Aldrich, catalog F1804-50UG), PDE4A (Abcam, catalog ab200383), PDE4C (Proteintech, catalog 21754-1-AP), PDE4B (Thermo Fisher Scientific, catalog 40-1400), and PDE4D (Sigma-Aldrich, catalog ABS22; Bethyl Laboratories, catalog A303-744A; Proteintech, catalog 12918-1-AP; and Thermo Fisher Scientific, catalog PA5-21590). The signals were developed with SuperSignal West Dura Substrate (Thermo Fisher Scientific, 34076). See complete unedited blots in the supplemental material.

### Subcellular fractionation.

Cells were lysed with HNMEK lysis buffer (20 mM HEPES pH 7.4, 50 mM NaCl, 2 mM MgCl_2_, 2 mM EDTA, 10 mM KCl, 50 nM EGTA, protease inhibitors) using a Dounce homogenizer. The nuclei and cell debris were removed from lysates by centrifugation at 750*g* for 10 minutes. The pellets were collected for organelle fractionation by centrifugation at 12,500*g* for 10 minutes, and supernatants were used as the cytoplasmic fraction. The pellets were lysed with RIPA buffer (50 mM Tris pH 8.0, 150 mM NaCl, 5 mM EDTA pH 8.0, 1% [v/v] NP-40, 0.5% (w/v) deoxycholate, protease inhibitor).

### Immunofluorescence.

Cells (1 × 10^5^) were cultured on coverslips in 12-well plates coated with fibronectin (Sigma-Aldrich) for 24 hours, and then fixed with 4% (v/v) paraformaldehyde for 20 minutes. Cells were washed twice with 1× PBS, and then permeabilized with 0.1% (v/v) Triton X-100 in PBS for 10 minutes. To block nonspecific antibody binding, 3% (w/v) BSA in PBS was used and primary antibodies were incubated at 4°C overnight. Primary antibodies against the following proteins were used: LAMP2 (Abcam, ab13524), mTOR (Cell Signaling Technology, catalog 2972), and PDE4D (Thermo Fisher Scientific, catalog PA5-21590). Secondary antibody was used Alexa Fluor 488– or 555–conjugated goat anti-rabbit or anti-mouse (1:500; Thermo Fisher Scientific, catalog A21428, A11017, and A21425) followed by washing with 1× PBS and mounted on microscope slides using ProlongGold with DAPI (Invitrogen, P36971). The mounted samples were imaged with an LSM 900 confocal microscope (Carl Zeiss). The images were quantified with Squassh in ImageJ (85).

### Cell size.

Cells (1 × 10^5^) were plated in triplicate in 12-well plates and were trypsinized and resuspended with 1× PBS after 48 hours. Cell number and size were measured using a Z2 Coulter Particle Count and Size Analyzer (Beckman Coulter) and processed using Z2 Accucomp software. Final binned-histogram plots were created with GraphPad Prism 9.

### Cell proliferation.

Cells (5 × 10^4^) were plated in triplicate in 12-well plates. Cells were prepared with Trypan blue solution (430166, Corning Inc.) at 1:1 ratio and then were counted after 24, 48, and 72 hours using a TC2 Automated Cell Counter (1450102, Bio-Rad).

### Clonogenic assay of cells in vitro.

Two hundred cells were seeded in 6-well plates and were incubated to form colonies for 14 days. The colonies were fixed with 20% (v/v) methanol and 0.5% (w/v) crystal violet (Sigma-Aldrich) and then were counted using ImageJ.

### Mouse xenografts.

MIA PaCa-2 cells (2 × 10^6^) expressing control (shGFP) or shPDE4D were subcutaneously injected into 6-week-old male NOD SCID mice (15 mice per group) purchased from the UT Southwestern mouse breeding core. When tumor volume reached 50 to 100 mm^3^, roflumilast (5 mg/kg, daily) and GEGR-7b (0.3 mg/kg, daily) were injected intraperitoneally into the mice. Tumor volume was measured every week with digital calipers and calculated by using the formula volume = length × width^2^/2.

### Immunohistochemistry.

Mouse xenograft pancreatic tumor tissues were formalin-fixed and paraffin-embedded. Sections were sliced at 5 μm thickness and placed on slides, and then deparaffinized and hydrated. Citrate buffer (10 mM citric acid, 0.05% [v/v] Tween 20, pH 6.0) was used for antigen retrieval, and then the slides were incubated in 3% (v/v) hydrogen peroxide, followed by primary antibodies overnight at 4°C. The slides were incubated with biotinylated secondary antibodies and VECTASTAIN ABC reagents (Vector Laboratories). Antigen signals were visualized using DAB (Vector Laboratories), and the slides were counterstained with hematoxylin (Sigma-Aldrich). The images were obtained using an LSM 900 (Carl Zeiss) and portions of the positive-stained areas were semiquantified with ImageJ. Human pancreatic cancer and paired benign tissue slides were obtained from the UT Southwestern Tissue Management Shared Resource. Immunohistochemical analysis was performed on a Dako Autostainer Link 48 system. Briefly, the slides were baked for 20 minutes at 60°C, and then deparaffinized and hydrated before the antigen retrieval step. Heat-induced antigen retrieval was performed at pH 9.0 for 20 minutes in a Dako PT Link. The tissue was incubated with a peroxidase block and then with an antibody (1:200 dilution) for 20 minutes. The staining was visualized using the Envision FLEX visualization system. The percentage and staining intensity of PDE4D-positive PDAC tumor cells or normal pancreatic ducts were recorded, using a grading scale of absent (0), weak (1+), moderate (2+), or strong (3+). An H-score was calculated as described previously (86). Antibodies against pS6 (Cell Signaling Technology, 4858) and PDE4D (Proteintech, 12918-1-AP) were used.

### Statistics.

The survival plots and expression of pancreatic adenocarcinoma patients were obtained from GEPIA (74) and the log-rank test was used for statistical analysis. The data are presented as mean ± standard deviation (SD). For immunohistochemical H-scores, mouse tumor growth and weight are presented as mean ± standard error of the mean (SEM). Statistical analysis was performed using the 2-tailed Student’s *t* test and 2-way ANOVA to compare 2 groups of independent experiments using GraphPad Prism 9. A *P* value of less than 0.05 was considered significant: *P < 0.05, **P < 0.01, ***P < 0.001, ****P < 0.0001.

### Study approval.

All mouse experiments were conducted according to approved guidelines at the Institutional Animal Care and Use Committee (IACUC) of UT Southwestern Medical Center.

Patients enrolled in the UT Southwestern study provided written consent, allowing the use of discarded surgical samples for research purposes according to the IRB-approved protocol. Human PDAC tissues were obtained from the UT Southwestern Tissue Repository under IRB-approved protocol 102010-051.

## Author contributions

MHJ designed and conducted the main experiments. GU, CL, and ZC provided technical assistance for the main experiments. JLJ conceived and supervised the project. MHJ and JLJ discussed and interpreted the results and wrote the manuscript.

## Supplementary Material

Supplemental data

## Version 1 07/10/2023

Electronic publication

## References

[1] Jewell JL (2013). Amino acid signalling upstream of mTOR. Nat Rev Mol Cell Biol.

[2] Gomes AP, Blenis J (2015). A nexus for cellular homeostasis: the interplay between metabolic and signal transduction pathways. Curr Opin Biotechnol.

[3] Zoncu R (2011). mTOR: from growth signal integration to cancer, diabetes and ageing. Nat Rev Mol Cell Biol.

[4] Valvezan AJ, Manning BD (2019). Molecular logic of mTORC1 signalling as a metabolic rheostat. Nat Metab.

[5] Saxton RA, Sabatini DM (2017). mTOR signaling in growth, metabolism, and disease. Cell.

[6] Hara K (2002). Raptor, a binding partner of target of rapamycin (TOR), mediates TOR action. Cell.

[7] Oshiro N (2004). Dissociation of raptor from mTOR is a mechanism of rapamycin-induced inhibition of mTOR function. Genes Cells.

[8] Kim D-H (2003). GbetaL, a positive regulator of the rapamycin-sensitive pathway required for the nutrient-sensitive interaction between raptor and mTOR. Mol Cell.

[9] Betz C, Hall MN (2013). Where is mTOR and what is it doing there?. J Cell Biol.

[10] Laplante M, Sabatini DM (2009). mTOR signaling at a glance. J Cell Sci.

[11] Tian T (2019). mTOR signaling in cancer and mTOR inhibitors in solid tumor targeting therapy. Int J Mol Sci.

[12] Hua H (2019). Targeting mTOR for cancer therapy. J Hematol Oncol.

[13] Bar-Peled L, Sabatini DM (2014). Regulation of mTORC1 by amino acids. Trends Cell Biol.

[14] Groenewoud MJ, Zwartkruis FJ (2013). Rheb and Rags come together at the lysosome to activate mTORC1. Biochem Soc Trans.

[15] Melick CH, Jewell JL (2020). Regulation of mTORC1 by upstream stimuli. Genes (Basel).

[16] Brady OA (2016). Rags to riches: amino acid sensing by the Rag GTPases in health and disease. Small GTPases.

[17] Yang H (2017). Mechanisms of mTORC1 activation by RHEB and inhibition by PRAS40. Nature.

[18] Manning BD, Cantley LC (2003). Rheb fills a GAP between TSC and TOR. Trends Biochem Sci.

[19] Inoki K (2003). Rheb GTPase is a direct target of TSC2 GAP activity and regulates mTOR signaling. Genes Dev.

[20] Sancak Y (2008). The Rag GTPases bind raptor and mediate amino acid signaling to mTORC1. Science.

[21] Nguyen TP (2017). Amino acid and small GTPase regulation of mTORC1. Cell Logist.

[22] Takahara T (2020). Amino acid-dependent control of mTORC1 signaling: a variety of regulatory modes. J Biomed Sci.

[23] Sancak Y (2010). Ragulator-Rag complex targets mTORC1 to the lysosomal surface and is necessary for its activation by amino acids. Cell.

[24] Bar-Peled L (2012). Ragulator is a GEF for the Rag GTPases that signal amino acid levels to mTORC1. Cell.

[25] Zoncu R (2011). mTORC1 senses lysosomal amino acids through an inside-out mechanism that requires the vacuolar H(+)-ATPase. Science.

[26] Bar-Peled L (2013). A Tumor suppressor complex with GAP activity for the Rag GTPases that signal amino acid sufficiency to mTORC1. Science.

[27] Tsun ZY (2013). The folliculin tumor suppressor is a GAP for the RagC/D GTPases that signal amino acid levels to mTORC1. Mol Cell.

[28] Wolfson RL (2017). KICSTOR recruits GATOR1 to the lysosome and is necessary for nutrients to regulate mTORC1. Nature.

[29] Shen K, Sabatini DM (2018). Ragulator and SLC38A9 activate the Rag GTPases through noncanonical GEF mechanisms. Proc Natl Acad Sci U S A.

[30] Jewell JL (2015). Metabolism. Differential regulation of mTORC1 by leucine and glutamine. Science.

[31] Meng D (2020). Glutamine and asparagine activate mTORC1 independently of Rag GTPases. J Biol Chem.

[32] Sassone-Corsi P (2012). The cyclic AMP pathway. Cold Spring Harb Perspect Biol.

[33] Weis WI, Kobilka BK (2018). The molecular basis of G protein-coupled receptor activation. Annu Rev Biochem.

[34] Hilger D (2018). Structure and dynamics of GPCR signaling complexes. Nat Struct Mol Biol.

[35] Yan K (2016). The cyclic AMP signaling pathway: exploring targets for successful drug discovery (Review). Mol Med Rep.

[36] Isobe K (2017). Systems-level identification of PKA-dependent signaling in epithelial cells. Proc Natl Acad Sci U S A.

[37] Soberg K (2017). Evolution of the cAMP-dependent protein kinase (PKA) catalytic subunit isoforms. PLoS One.

[38] Zhang H (2020). Complex roles of cAMP-PKA-CREB signaling in cancer. Exp Hematol Oncol.

[39] Sapio L (2014). Targeting protein kinase A in cancer therapy: an update. EXCLI J.

[40] Autenrieth K (2016). Defining A-kinase anchoring protein (AKAP) specificity for the protein kinase a subunit RI (PKA-RI). Chembiochem.

[41] Ercu M, Klussmann E (2018). Roles of A-kinase anchoring proteins and phosphodiesterases in the cardiovascular system. J Cardiovasc Dev Dis.

[42] Keravis T, Lugnier C (2012). Cyclic nucleotide phosphodiesterase (PDE) isozymes as targets of the intracellular signalling network: benefits of PDE inhibitors in various diseases and perspectives for future therapeutic developments. Br J Pharmacol.

[43] Hsien Lai S (2020). PDE4 subtypes in cancer. Oncogene.

[44] Peng T (2018). Inhibitors of phosphodiesterase as cancer therapeutics. Eur J Med Chem.

[45] Conti M (2003). Cyclic AMP-specific PDE4 phosphodiesterases as critical components of cyclic AMP signaling. J Biol Chem.

[46] Fertig BA, Baillie GS (2018). PDE4-mediated cAMP signalling. J Cardiovasc Dev Dis.

[47] Richter W (2005). Splice variants of the cyclic nucleotide phosphodiesterase PDE4D are differentially expressed and regulated in rat tissue. Biochem J.

[48] Wang L (2015). UCR1C is a novel activator of phosphodiesterase 4 (PDE4) long isoforms and attenuates cardiomyocyte hypertrophy. Cell Signal.

[49] Beard MB (2000). UCR1 and UCR2 domains unique to the cAMP-specific phosphodiesterase family form a discrete module via electrostatic interactions. J Biol Chem.

[50] Mika D, Conti M (2016). PDE4D phosphorylation: a coincidence detector integrating multiple signaling pathways. Cell Signal.

[51] Liu F (2019). High expression of PDE4D correlates with poor prognosis and clinical progression in pancreaticductal adenocarcinoma. J Cancer.

[52] Pullamsetti SS (2013). Phosphodiesterase-4 promotes proliferation and angiogenesis of lung cancer by crosstalk with HIF. Oncogene.

[53] Mishra RR (2018). Reactivation of cAMP pathway by PDE4D inhibition represents a novel druggable axis for overcoming tamoxifen resistance in ER-positive breast cancer. Clin Cancer Res.

[54] Powers GL (2015). Phosphodiesterase 4D inhibitors limit prostate cancer growth potential. Mol Cancer Res.

[55] Lin C-H (2016). Recent advances using phosphodiesterase 4 (PDE4) inhibitors to treat inflammatory disorders: animal and clinical studies. Curr Drug Ther.

[56] Park SJ (2012). Resveratrol ameliorates aging-related metabolic phenotypes by inhibiting cAMP phosphodiesterases. Cell.

[57] Jewell JL (2019). GPCR signaling inhibits mTORC1 via PKA phosphorylation of Raptor. Elife.

[58] Phillips JE (2020). Inhaled phosphodiesterase 4 (PDE4) inhibitors for inflammatory respiratory diseases. Front Pharmacol.

[59] Li H (2018). Phosphodiesterase-4 inhibitors for the treatment of inflammatory diseases. Front Pharmacol.

[60] Kim J, Guan KL (2019). mTOR as a central hub of nutrient signalling and cell growth. Nat Cell Biol.

[61] Houslay MD (2007). cAMP-specific phosphodiesterase-4 enzymes in the cardiovascular system: a molecular toolbox for generating compartmentalized cAMP signaling. Circ Res.

[62] Ong WK (2009). The role of the PDE4D cAMP phosphodiesterase in the regulation of glucagon-like peptide-1 release. Br J Pharmacol.

[63] Bolger GB (2020). Dominant-negative attenuation of cAMP-selective phosphodiesterase PDE4D action affects learning and behavior. Int J Mol Sci.

[64] Zhang S (2021). AKAP13 couples GPCR signaling to mTORC1 inhibition. PLoS Genet.

[65] Smith FD (2011). Discovery of cellular substrates for protein kinase A using a peptide array screening protocol. Biochem J.

[66] Lutz W (2001). Phosphorylation of centrin during the cell cycle and its role in centriole separation preceding centrosome duplication. J Biol Chem.

[67] Artimo P (2012). ExPASy: SIB bioinformatics resource portal. Nucleic Acids Res.

[68] Mackenzie SJ, Houslay MD (2000). Action of rolipram on specific PDE4 cAMP phosphodiesterase isoforms and on the phosphorylation of cAMP-response-element-binding protein (CREB) and p38 mitogen-activated protein (MAP) kinase in U937 monocytic cells. Biochem J.

[69] Rabe KF (2011). Update on roflumilast, a phosphodiesterase 4 inhibitor for the treatment of chronic obstructive pulmonary disease. Br J Pharmacol.

[70] Bruno O (2011). GEBR-7b, a novel PDE4D selective inhibitor that improves memory in rodents at non-emetic doses. Br J Pharmacol.

[71] Long X (2005). Rheb binds and regulates the mTOR kinase. Curr Biol.

[72] Blackman BE (2011). PDE4D and PDE4B function in distinct subcellular compartments in mouse embryonic fibroblasts. J Biol Chem.

[73] Laplante M, Sabatini DM (2013). Regulation of mTORC1 and its impact on gene expression at a glance. J Cell Sci.

[74] Tang Z (2017). GEPIA: a web server for cancer and normal gene expression profiling and interactive analyses. Nucleic Acids Res.

[75] Faller WJ (2015). mTORC1-mediated translational elongation limits intestinal tumour initiation and growth. Nature.

[76] Engelman JA (2008). Effective use of PI3K and MEK inhibitors to treat mutant Kras G12D and PIK3CA H1047R murine lung cancers. Nat Med.

[77] Valvezan AJ (2017). mTORC1 couples nucleotide synthesis to nucleotide demand resulting in a targetable metabolic vulnerability. Cancer Cell.

[78] Rao R (2016). The G protein Gα_s_ acts as a tumor suppressor in sonic hedgehog signaling-driven tumorigenesis. Cell Cycle.

[79] Iglesias-Bartolome R (2015). Inactivation of a Gα(s)-PKA tumour suppressor pathway in skin stem cells initiates basal-cell carcinogenesis. Nat Cell Biol.

[80] He X (2014). The G protein α subunit Gα_s_ is a tumor suppressor in Sonic hedgehog-driven medulloblastoma. Nat Med.

[81] Fernandez-Araujo A (2015). Key role of phosphodiesterase 4A (PDE4A) in autophagy triggered by yessotoxin. Toxicology.

[82] Kim DU (2019). Phosphodiesterase 4B is an effective therapeutic target in colorectal cancer. Biochem Biophys Res Commun.

[83] Stark C (2006). BioGRID: a general repository for interaction datasets. Nucleic Acids Res.

[84] Sriram K, Insel PA (2018). G protein-coupled receptors as targets for approved drugs: how many targets and how many drugs?. Mol Pharmacol.

[85] Rizk A (2014). Segmentation and quantification of subcellular structures in fluorescence microscopy images using Squassh. Nat Protoc.

[86] McCarty KS (1986). Use of a monoclonal anti-estrogen receptor antibody in the immunohistochemical evaluation of human tumors. Cancer Res.

