# Supplementary material for: Inhibition of phosphodiesterase 4D suppresses mTORC1 signaling and pancreatic cancer growth
Source: JCI Insight. 2023 Jul 10;8(13):e158098. doi: 10.1172/jci.insight.158098 (PMC10371348; doi:10.1172/jci.insight.158098)
Supplement: Supplemental data [file jciinsight-8-158098-s006.pdf]

Supplemental Figures

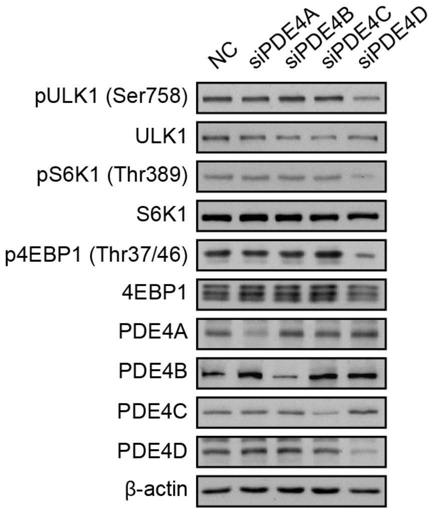

Supplemental Figure 1 (Related to Figure 1)

PDE4A-C do not regulate mTORC1 activity. Depletion of PDE4A-C isoforms do not alter mTORC1 signaling. siRNAs for PDE4A, PDE4B, PDE4C, and PDE4D were transfected in HEK293A cells. After 48h cell lysates were assessed by immunoblotting for mTORC1 activity (pULK1 (Ser758), pS6K1 (Thr389), p4EBP1 (Thr37/46)). S6K1, ULK1, 4EBP1, PDE4A, PDE4B, PDE4C, PDE4D, β-actin are controls.

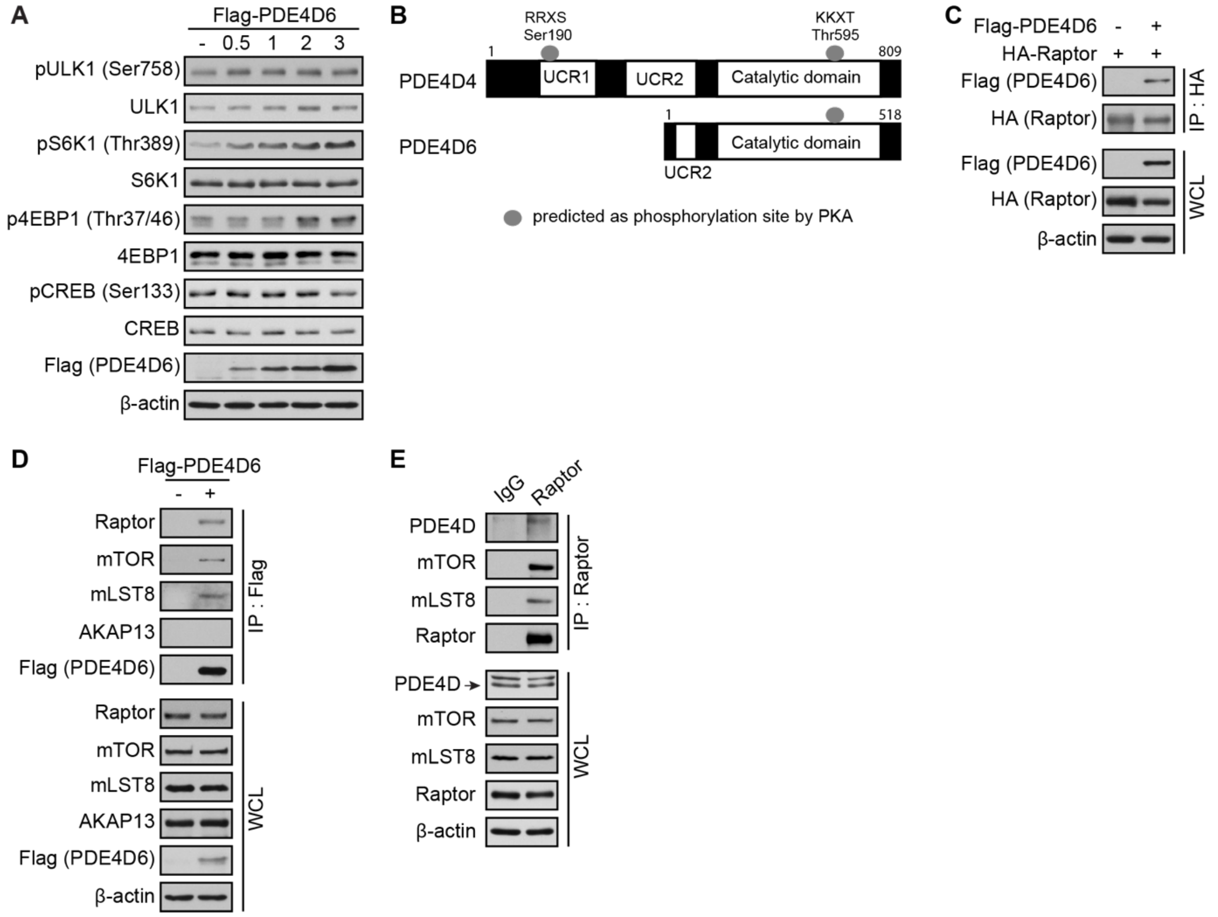

Supplemental Figure 2 (Related to Figure 1)

PDE4D interacts with and regulates mTORC1. (**A**) PDE4D regulates mTORC1 signaling. HEK293A cells were transfected with Flag-tagged Empty vector or Flag-tagged PDE4D6 for 48h and then mTORC1 activity was assessed by pULK1 (Ser758), pS6K1(Thr389), and p4EBP1(Thr37/46). ULK1, S6K1, 4EBP1, p-CREB (Ser133) (measure PKA activation), CREB, Flag (PDE4D6), and β-actin are controls. (**B**) Schematic of PDE4D domains and predicted PKA phosphorylation sites. (**C-E**) PDE4D binds to mTORC1. (**C**) Cells were transfected with HA-tagged Raptor and Flag-tagged PDE4D6 and then immunoprecipitated (IP) with HA antibody (Raptor). Flag (PDE4D6), HA (Raptor), and β-actin were analyzed by Western blot. WCL: Whole cell lysate. (**D**) Cells were transfected with Flag-tagged PDE4D6 and then immunoprecipitated (IP) with Flag antibody (PDE4D6). Raptor, mTOR, mLST8, AKAP13, Flag (PDE4D6), β-actin were analyzed by Western blot. WCL: Whole cell lysate. (**E**) Cell lysates were immunoprecipitated (IP) with IgG or Raptor antibody. PDE4D, mTOR, mLST8, Raptor, and β-actin were analyzed by western blot. WCL: Whole cell lysate.

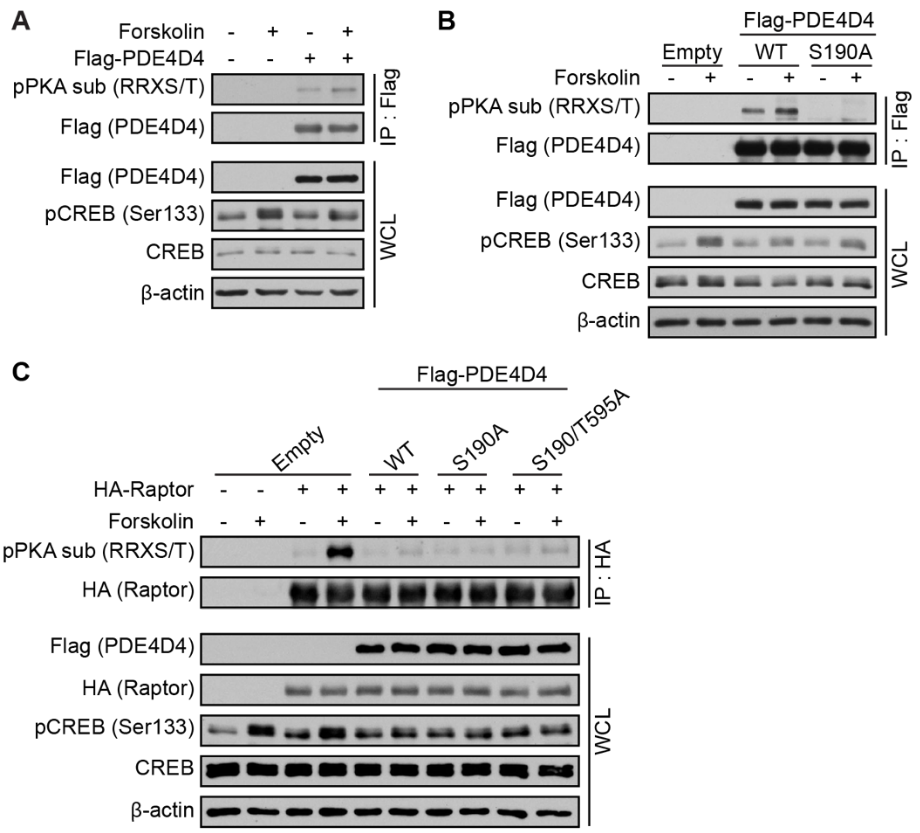

Supplemental Figure 3 (Related to Figure 2)

PDE4D phosphorylation by PKA does not alter Raptor Ser791 phosphorylation. (**A**) PDE4D is phosphorylated by PKA. HEK293A cells were transfected with Flag-tagged Empty vector or Flag-tagged PDE4D4 for 48h and stimulated with forskolin (10 μM) for 1h. Lysates were immunoprecipitated (IP) with Flag antibody (PDE4D4) and assessed by immunoblotting for pPKA substrate (RRXS/T) antibody. Flag (PDE4D4), pCREB (Ser133) (measure of PKA activation), CREB, and β-actin are controls. WCL: Whole cell lysates. (**B**) PKA phosphorylates PDE4D at Ser190. Flag-tagged Empty vector, Flag-tagged PDE4D4 wild type (WT), and Flag-tagged PDE4D4 Ser190 mutated to Ala190 (S190A) constructs were transfected for 48h, and stimulated with forskolin (10 μM) for 1h in HEK293A cells. Lysates were immunoprecipitated with Flag antibody (PDE4D4) and assessed by immunoblotting for pPKA substrate (RRXS/T) antibody. Flag (PDE4D4), pCREB (Ser133) (measure of PKA activation), CREB, and β-actin are controls. WCL: Whole cell lysates. (**C**) PKA phosphorylation by PDE4D does not alter Raptor Ser791 phosphorylation. Flag-tagged Empty vector, HA-tagged Empty vector, HA-tagged Raptor, Flag-tagged PDE4D4 wild type (WT), Flag-tagged PDE4D4 Ser190 mutated to Ala190 (S190A), or Flag-tagged PDE4D4 Ser190 mutated to Ala190/Thr595 mutated to Ala595 (S190A/T595A) were expressed in HEK293A cells. After 48h cells were treated with forskolin (10 μM) for 1h and then lysates immunoprecipitated (IP) with HA antibody (Raptor) and assessed by immunoblotting for pPKA substrate (RRXS*/T*) antibody. Flag (PDE4D4), HA (Raptor), pCREB (Ser133) (measure of PKA activation), CREB, and β-actin are controls. WCL: Whole cell lysates.

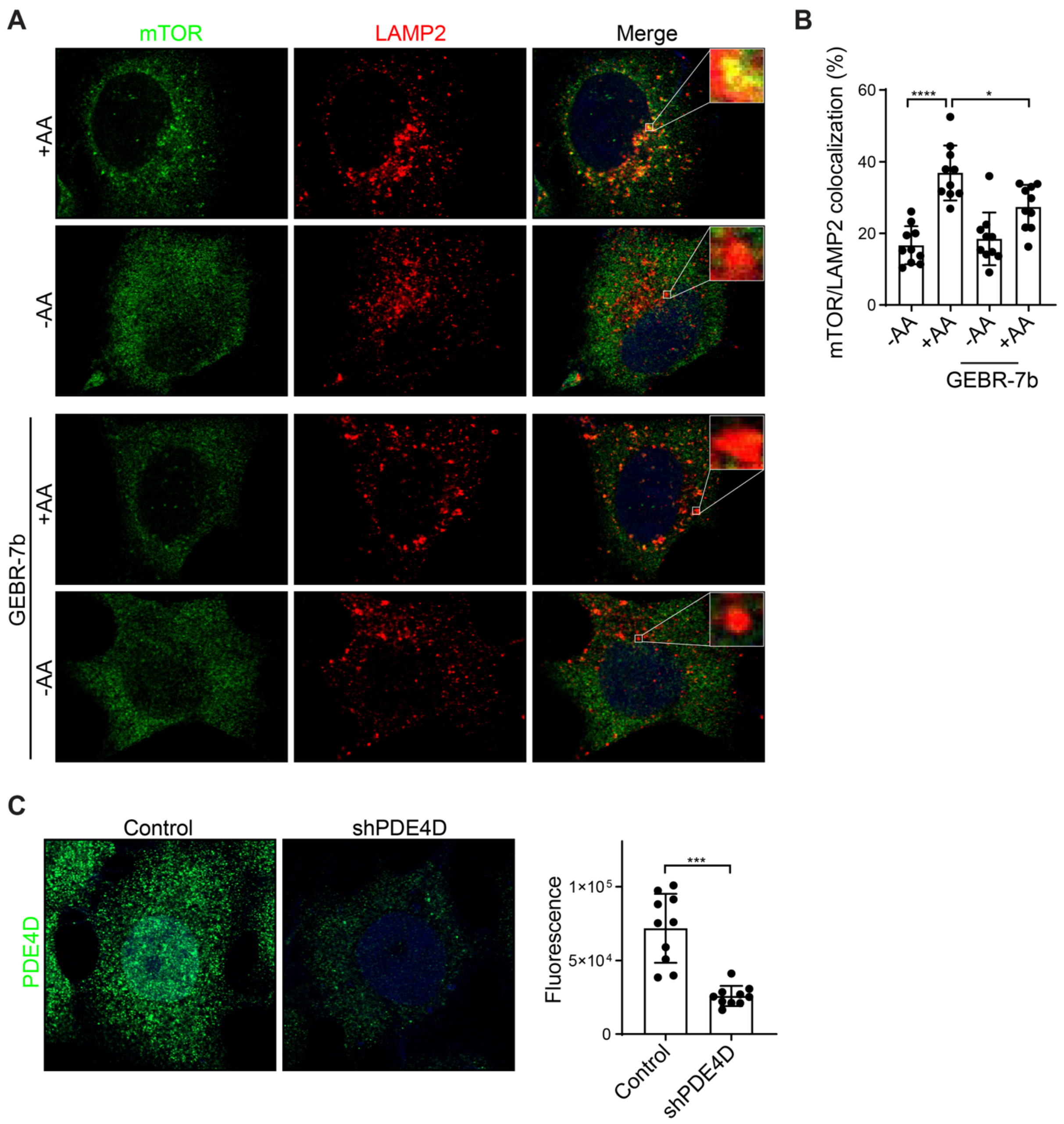

Supplemental Figure 4 (Related to Figure 3)

PDE4D promotes mTORC1 lysosomal localization. (**A-B**) PDE4D inhibition blocks amino acid-induced mTORC1 lysosomal localization. HEK293A cells were starved in in amino acid free media for 2h, and then stimulated with or without amino acids for 2h, with or without GEBR-7b (20 μg/ml). Immunofluorescent experiments were performed with mTOR or LAMP2 antibodies. Representative images obtained under LSM900 confocal microscope with 100X objective. (**B**) Ten images per group in (A) were quantified using Squassh. The data represent mean ± SD. For statistical analysis, two-way ANOVA with Tukey's test for multiple comparisons was performed. *P<0.05 and ****P<0.0001. (**C**) PDE4D localizes in the cytoplasm. To validate an endogenous PDE4D, immunofluorescent experiments were performed with a PDE4D antibody in control or PDE4D knocked down in HEK293A cells. Representative images obtained as described in (A) and intensity were quantified using Image J. The data represent mean ± SD. ***P<0.001. For statistical analysis, Student's t test was performed.

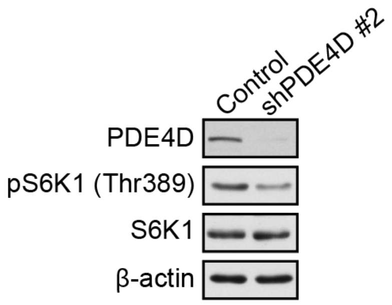

Supplemental Figure 5 (Related to Figure 4)

PDE4D depletion inhibits mTORC1 activity. MIA PaCa-2 cells expressing shGFP (Control) or shRNA targeting PDE4D (shPDE4D #2) were generated. Lysates were assessed for mTORC1 activity by immunoblotting for pS6K1 (Thr389). S6K1 and β-actin are controls.

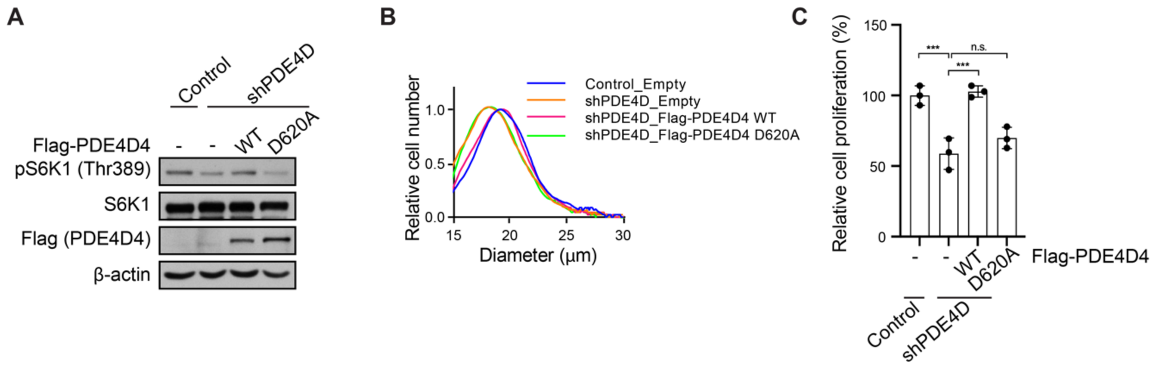

Supplemental Figure 6 (Related to Figure 4)

PDE4D controls pancreatic cancer cell growth *in vitro*. (**A**) mTORC1 activity is rescued in PDE4D depleted cells by re-introducing Flag-tagged PDE4D. Wildtype or an inactive PDE4D mutant (D620A) were expressed in shPDE4D MIA PaCa-2 cells. mTORC1 activity was analyzed as in by immunoblotting for pS6K1 (Thr389). S6K1, Flag (PDE4D), and β-actin are controls. (**B**). PDE4D regulates cell size. Cells were measured in Z2 Coulter Particle Count and Size Analyzer. For statistical analysis, a one-way ANOVA with Dunnett's's test for multiple comparisons was performed. Control_Empty vs shPDE4D_Empty $P<0.01$, Control_Empty vs shPDE4D_Flag-PDE4D4 WT n.s., Control_Empty vs shPDE4D_Flag-PDE4D4 D620A $P<0.05$. (**C**) PDE4D regulates cell proliferation. Data represents mean ± SD. For statistical analysis, two-way ANOVA with Tukey's test for multiple comparisons was performed. ***$P< 0.001$. n.s.: not significant.

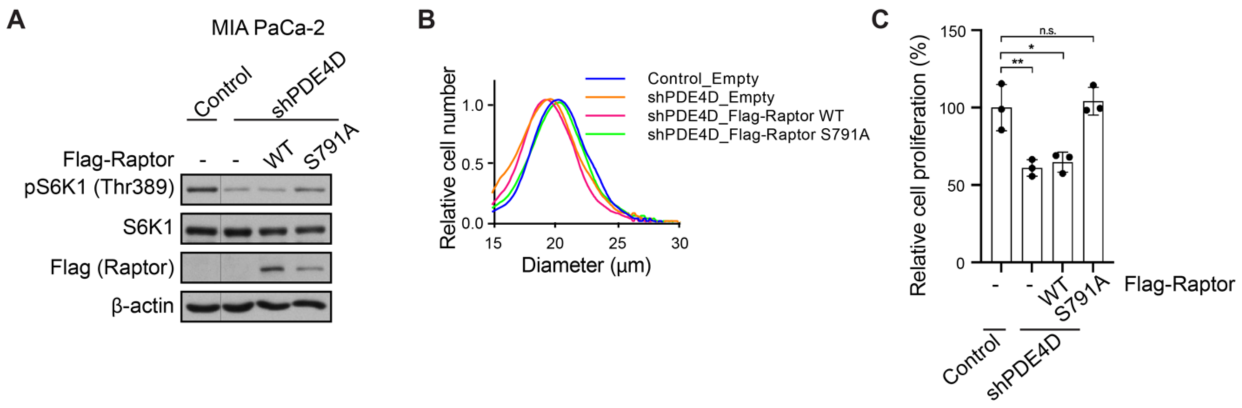

Supplemental Figure 7 (Related Figure 4)

Raptor Ser791 phosphorylation controls pancreatic cancer cell growth *in vitro*. (**A**) mTORC1 activity is rescued in PDE4D depleted cells by re-introducing Flag-tagged Raptor Ser791 mutant (Ser791 mutated to Ala791, S791A). Wildtype or Flag-tagged Raptor S791A were expressed in shPDE4D MIA PaCa-2 cells. mTORC1 activity was analyzed as in by immunoblotting for pS6K1 (Thr389). S6K1, Flag (PDE4D), and β-actin are controls. (**B**). PDE4D and Raptor Ser791 phosphorylation regulates cell size. MIA PaCa-2 cells were measured in Z2 Coulter Particle Count and Size Analyzer. For statistical analysis, a one-way ANOVA with Dunnett's test for multiple comparisons was performed. Control_Empty vs shPDE4D_Empty $P<0.001$, Control_Empty vs shPDE4D_Flag-Raptor WT $P<0.001$, and Control_Empty vs shPDE4D_Flag-Raptor S791A $P<0.5$. (**C**) PDE4D and Raptor Ser791 phosphorylation regulates MIA PaCa-2 cell proliferation. Data represents mean ± SD. For statistical analysis, two-way ANOVA with Tukey's test for multiple comparisons was performed. *$P< 0.05$. n.s.: not significant.

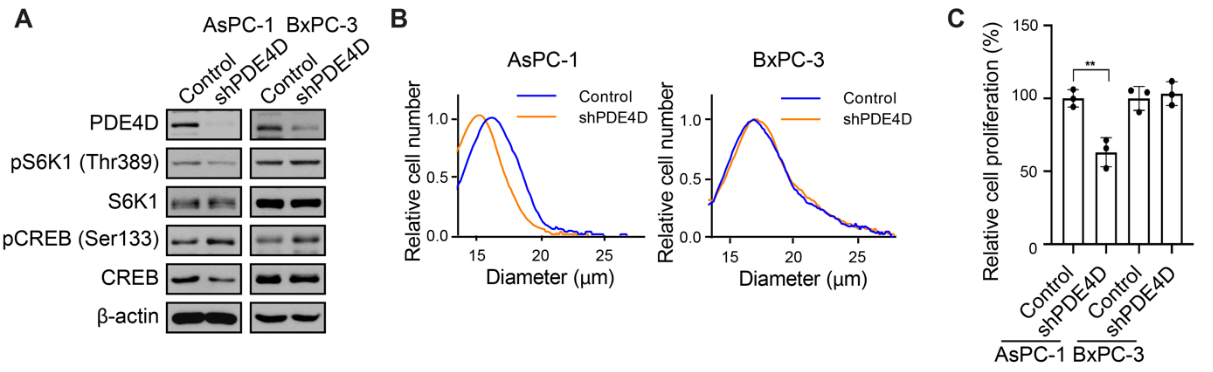

Supplemental Figure 8 (Related to Figure 4)

PDE4D regulates mTORC1 activity and pancreatic cancer growth when KRAS is mutated. (**A**) Depletion of PDE4D inhibits mTORC1 activity in KRAS mutant cells. PDE4D was knocked down in KRAS mutant (G12D) AsPC-1 or KRAS wild type (WT) BxPC-3 pancreatic cancer cells. Cell lysates were assessed by immunoblotting for mTORC1 activity was analyzed as in by immunoblotting for pS6K1 (Thr389). PDE4D, S6K1, pCREB (Ser133) (measure of PKA activation), CREB, and β-actin are controls. (**B**) PDE4D regulates cell size in KRAS mutant cells. Cells were measured in Z2 Coulter Particle Count and Size Analyzer. For statistical analysis, Student's t test was performed. AsPC-1 Control vs shPDE4D $P<0.0001$ and BxPC-3 Control vs shPDE4D n.s. (**C**) PDE4D regulates cell proliferation in KRAS mutant cells. Data represents mean ± SD. For statistical analysis, two-way ANOVA with Tukey's test for multiple comparisons was performed. $^{**}P<0.05$. not significant.

Case No.	Tissue No.	PDAC %	PDAC Intensity	PDAC H-score	NL duct %	NL duct Intensity	NL duct H-score
1	20981	100	2	200	25	1	25
2	24653	100	3	300	25	1	25
3	24846	90	3	270	10	1	10
4	24855	100	3	300	10	1	10
5	25221	80	2	160	50	1	50
6	25477	80	2	160	Fat, no pancreatic tissue present		
7	25833	70	3	210	small bowel, no pancreatic tissue		
8	26093	80	1	80	Fat, no pancreatic tissue present		
9	27438	80	3	240	Fat, no pancreatic tissue present		
10	28576	50	2	100	50	1	50
11	29585	80	3	240	25	1	25
12	30114	80	3	240	no normal tissue, both are cance		
13	30174	80	2	160	Fat, no pancreatic tissue present		
14	31714	90	3	270	50	1	50
15	32218	90	2	180	20	2	40
16	32277	80	2	160	10	1	10
17	33494	90	3	270	50	2	100
18	35553	90	3	270	10	2	20
19	33608	70	2	140	10	1	10
n				19			13
Average				208			33
SE				15			7

Supplemental Table 1 (Related to Figure 4)

The percentage and staining intensity of PDE4D in PDAC and normal pancreatic duct tissues. H-score was given as the percentage of positive cells multiplied by a score value corresponding to the intensity of positivity. 0 = negative, 1 = weak, 2 = moderate, and 3 = strong. The score on each case ranged from 0 (negative) to 300 (strong and diffuse positively). PDAC: Pancreatic ductal adenocarcinoma and NL duct: Normal duct

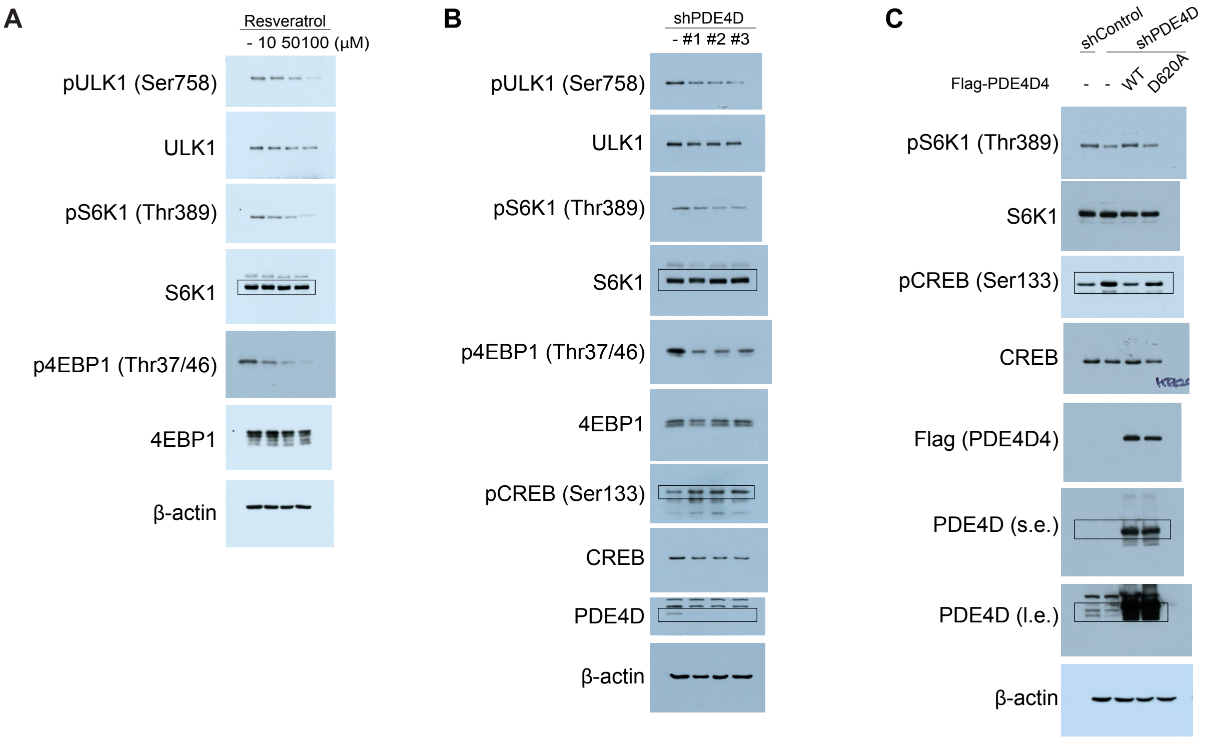
A
Resveratrol
- 10 50 100 (µM)
pULK1 (Ser758)
ULK1
pS6K1 (Thr389)
S6K1
p4EBP1 (Thr37/46)
4EBP1
β-actin
B
shPDE4D
- #1 #2 #3
pULK1 (Ser758)
ULK1
pS6K1 (Thr389)
S6K1
p4EBP1 (Thr37/46)
4EBP1
pCREB (Ser133)
CREB
PDE4D
β-actin
C
shControl
shPDE4D
Flag-PDE4D4
- - WT D620A
pS6K1 (Thr389)
S6K1
pCREB (Ser133)
CREB
Flag (PDE4D4)
PDE4D (s.e.)
PDE4D (l.e.)
β-actin

Figure 1

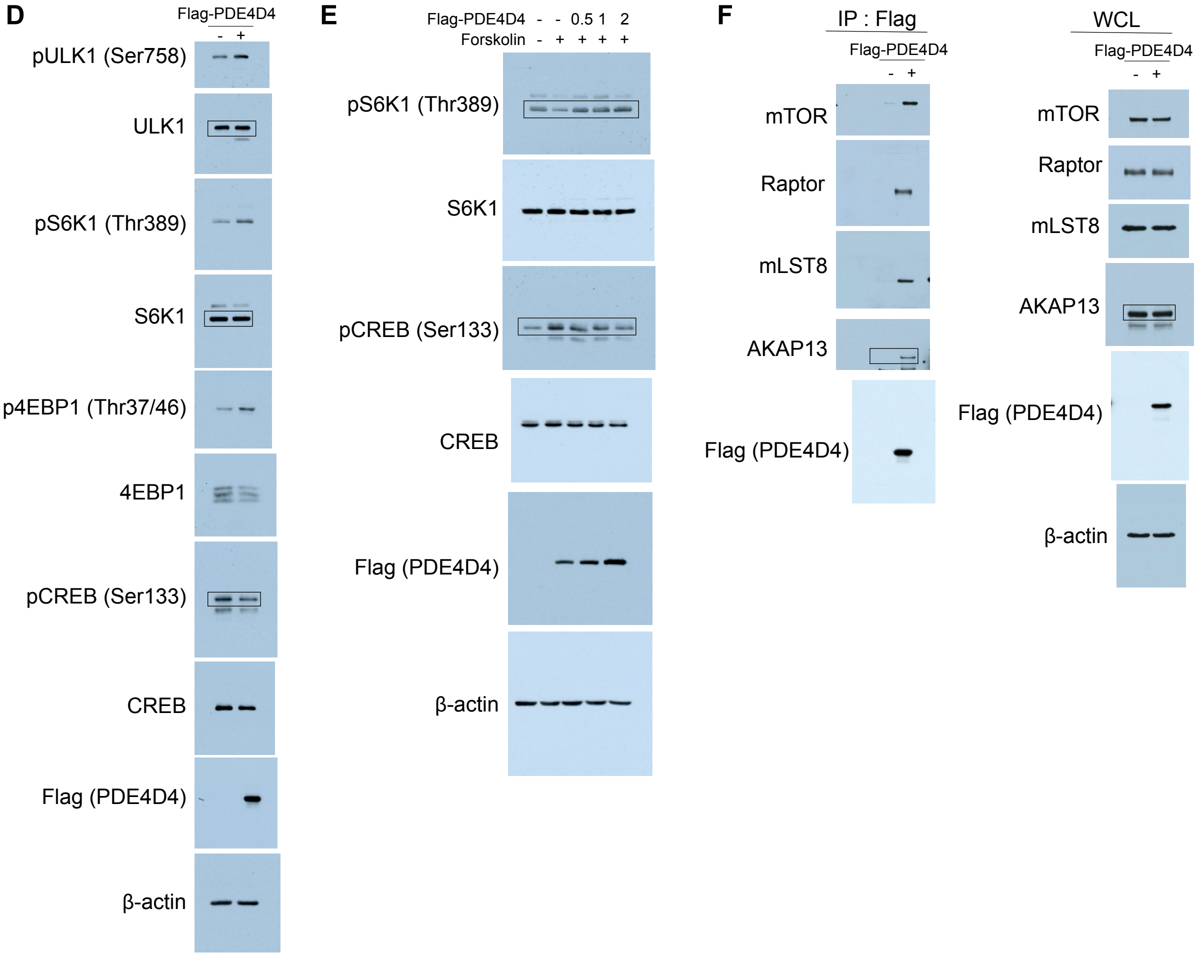
D
Flag-PDE4D4
- +
pULK1 (Ser758)
ULK1
pS6K1 (Thr389)
S6K1
p4EBP1 (Thr37/46)
4EBP1
pCREB (Ser133)
CREB
Flag (PDE4D4)
β-actin
E
Flag-PDE4D4 - - 0.5 1 2
Forskolin - + + + +
pS6K1 (Thr389)
S6K1
pCREB (Ser133)
CREB
Flag (PDE4D4)
β-actin
F
IP : Flag
Flag-PDE4D4
- +
mTOR
Raptor
mLST8
AKAP13
Flag (PDE4D4)
WCL
Flag-PDE4D4
- +
mTOR
Raptor
mLST8
AKAP13
Flag (PDE4D4)
β-actin

Figure 1

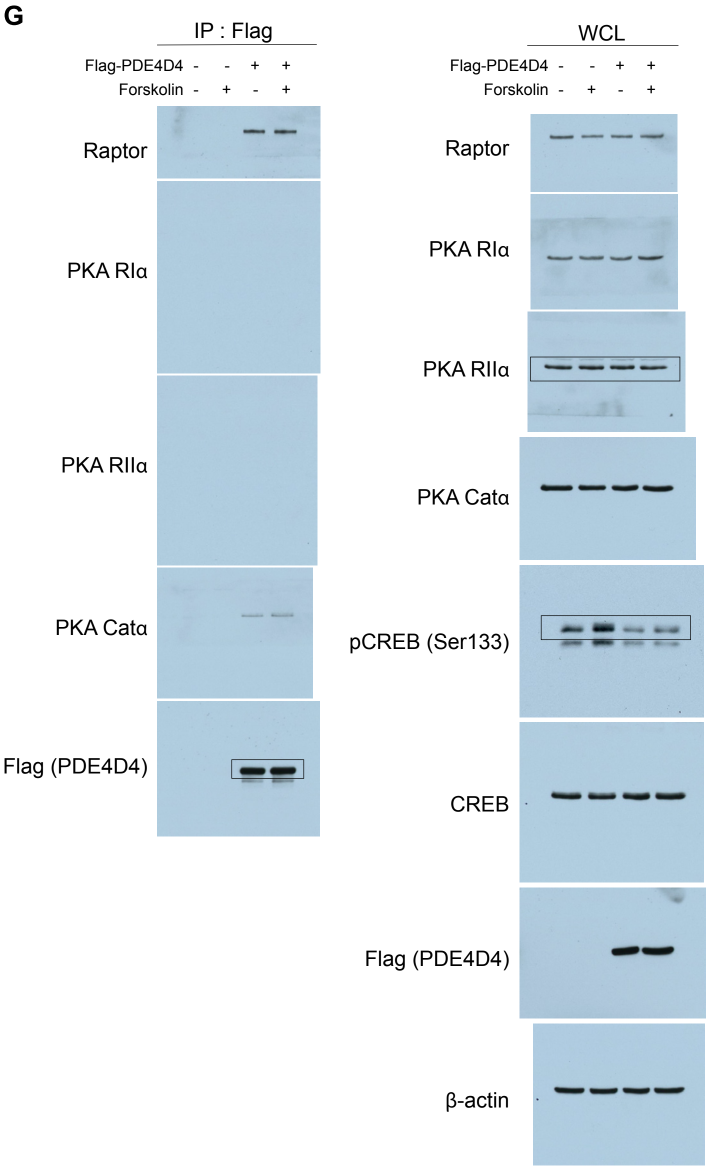
G
IP : Flag
WCL
Flag-PDE4D4 - - + +
Forskolin - + - +
Flag-PDE4D4 - - + +
Forskolin - + - +
Raptor
PKA RIα
PKA RIIα
PKA Catα
Flag (PDE4D4)
Raptor
PKA RIα
PKA RIIα
PKA Catα
pCREB (Ser133)
CREB
Flag (PDE4D4)
β-actin

Figure 1

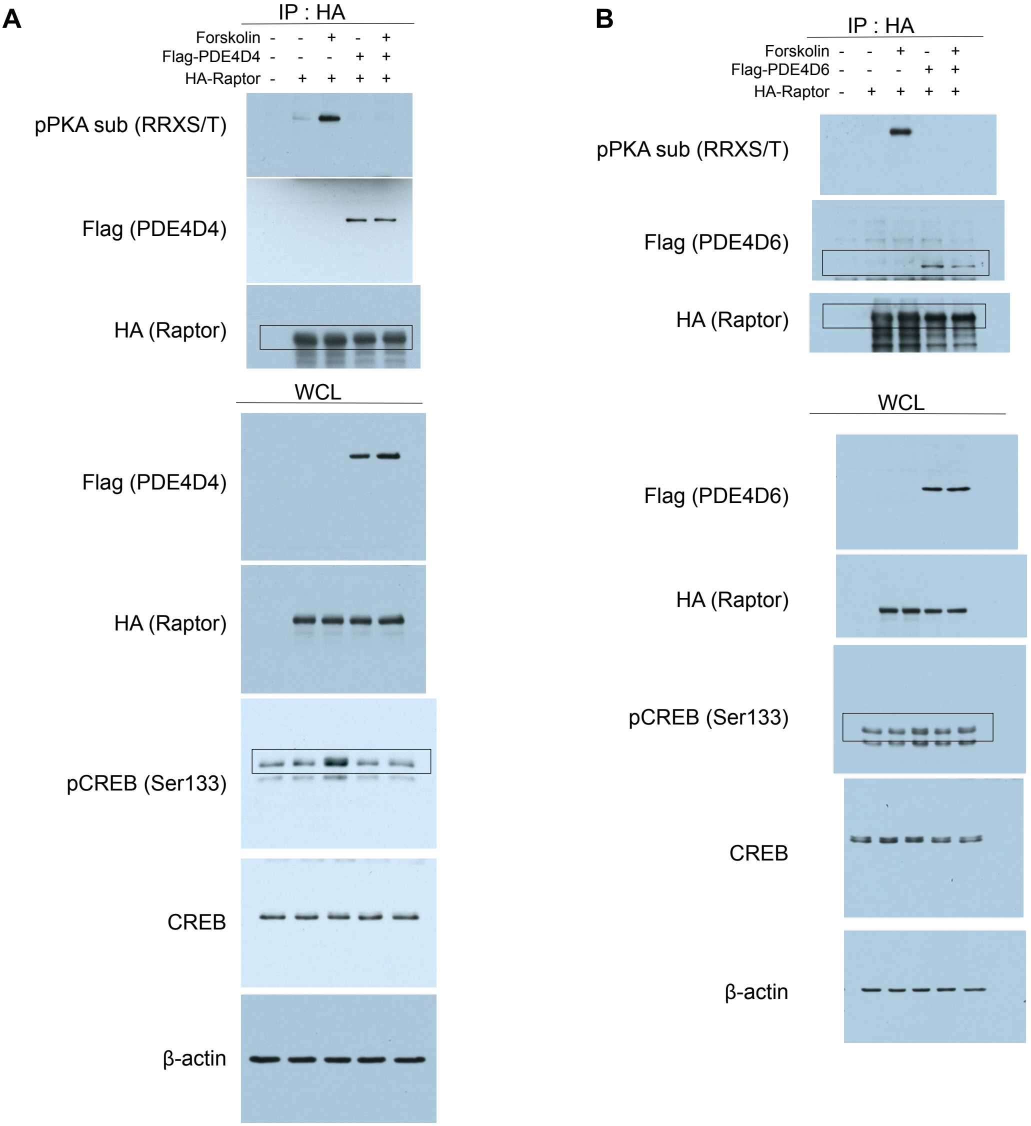
A
IP : HA
Forskolin - - + - +
Flag-PDE4D4 - - - + +
HA-Raptor - + + + +
pPKA sub (RRXS/T)
Flag (PDE4D4)
HA (Raptor)
WCL
Flag (PDE4D4)
HA (Raptor)
pCREB (Ser133)
CREB
β-actin
B
IP : HA
Forskolin - - + - +
Flag-PDE4D6 - - - + +
HA-Raptor - + + + +
pPKA sub (RRXS/T)
Flag (PDE4D6)
HA (Raptor)
WCL
Flag (PDE4D6)
HA (Raptor)
pCREB (Ser133)
CREB
β-actin

Figure 2

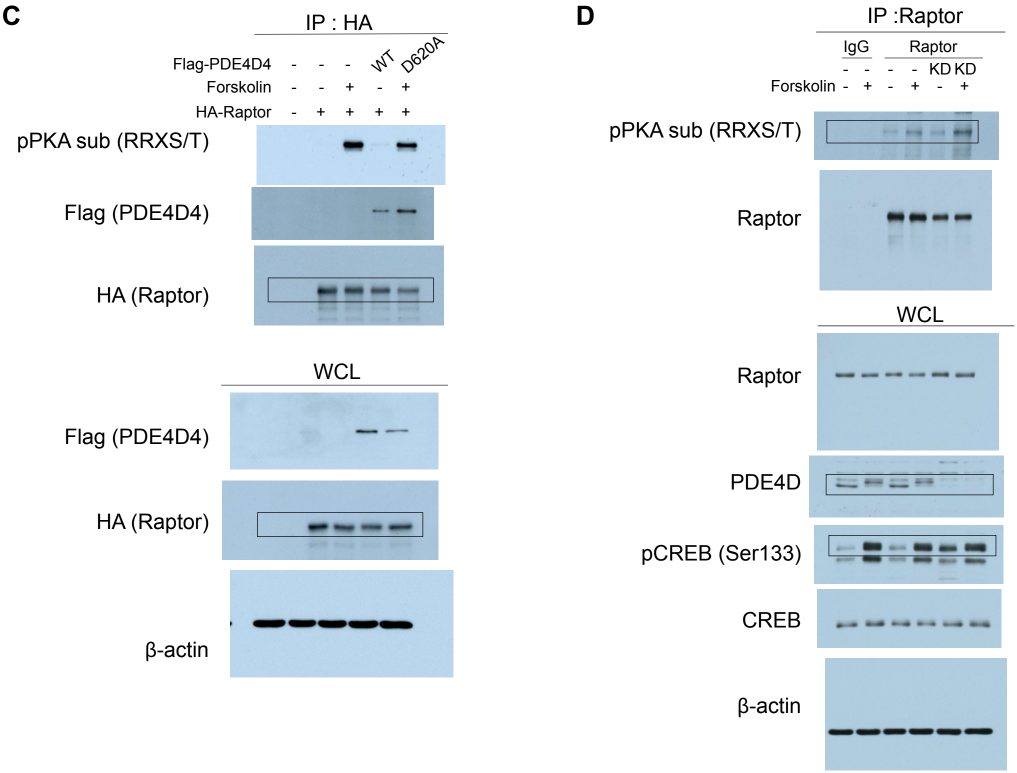
C
IP : HA
Flag-PDE4D4 - - - WT D620A
Forskolin - - + - +
HA-Raptor - + + + +
pPKA sub (RRXS/T)
Flag (PDE4D4)
HA (Raptor)
WCL
Flag (PDE4D4)
HA (Raptor)
β-actin
D
IP :Raptor
IgG
Raptor
- - - - KD KD
Forskolin - + - + - +
pPKA sub (RRXS/T)
Raptor
WCL
Raptor
PDE4D
pCREB (Ser133)
CREB
β-actin

Figure 2

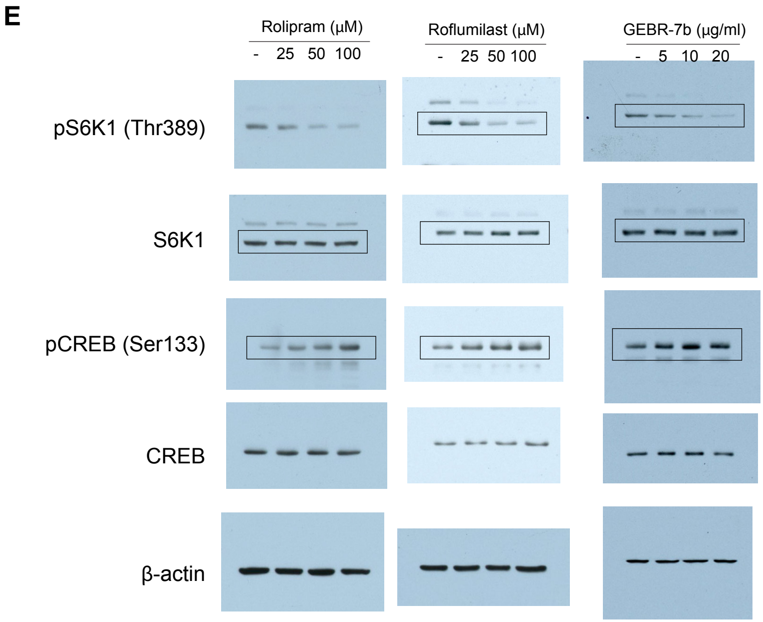
E
Rolipram (μM)
- 25 50 100
Roflumilast (μM)
- 25 50 100
GEBR-7b (μg/ml)
- 5 10 20
pS6K1 (Thr389)
S6K1
pCREB (Ser133)
CREB
β-actin

Figure 2

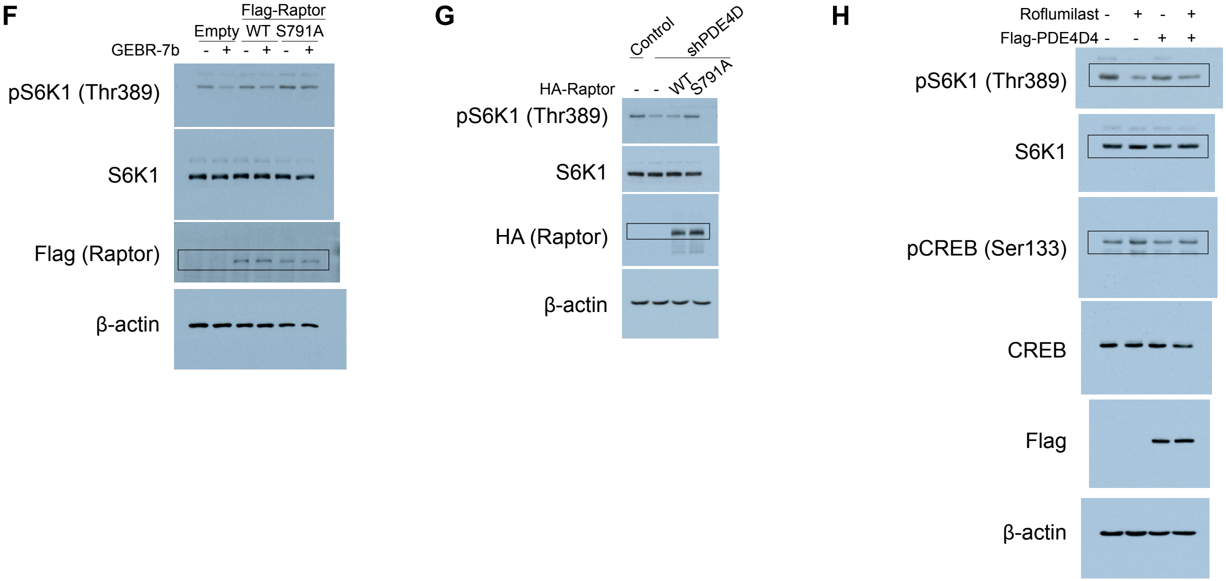

F
Flag-Raptor
Empty WT S791A
GEBR-7b - + - + - +
pS6K1 (Thr389)
S6K1
Flag (Raptor)
β-actin
G
Control
shPDE4D
HA-Raptor - - WT S791A
pS6K1 (Thr389)
S6K1
HA (Raptor)
β-actin
H
Roflumilast - + - +
Flag-PDE4D4 - - + +
pS6K1 (Thr389)
S6K1
pCREB (Ser133)
CREB
Flag
β-actin

Figure 2

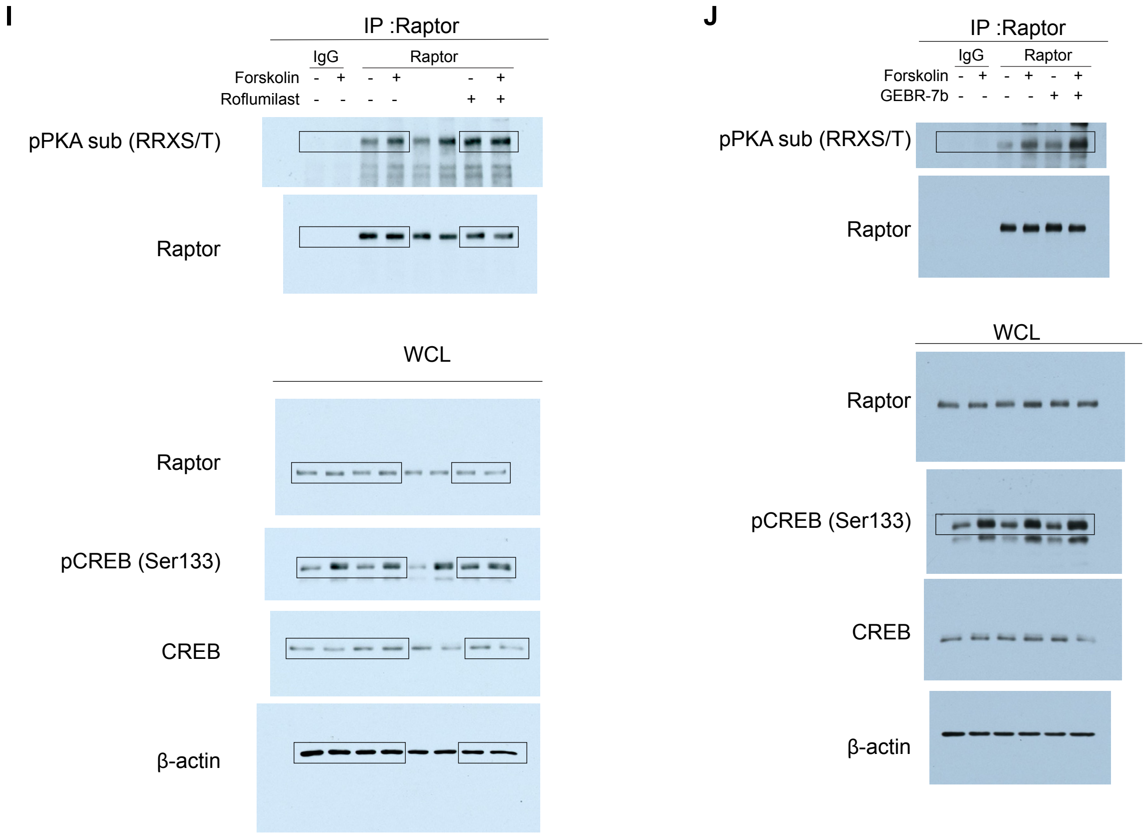
I
IP :Raptor
IgG
Raptor
Forskolin - + - + - +
Roflumilast - - - - + +
pPKA sub (RRXS/T)
Raptor
WCL
Raptor
pCREB (Ser133)
CREB
β-actin
J
IP :Raptor
IgG
Raptor
Forskolin - + - + - +
GEBR-7b - - - - + +
pPKA sub (RRXS/T)
Raptor
WCL
Raptor
pCREB (Ser133)
CREB
β-actin

Figure 2

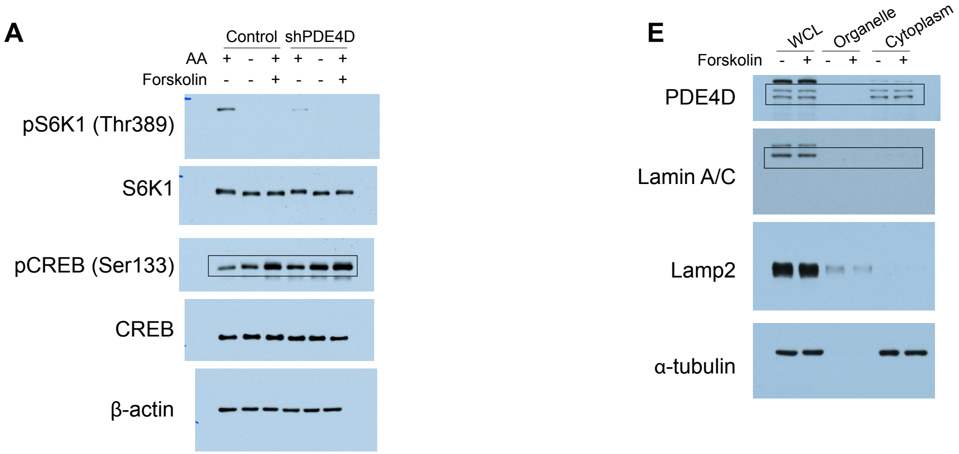
A
Control
shPDE4D
AA + - + + - +
Forskolin - - + - - +
pS6K1 (Thr389)
S6K1
pCREB (Ser133)
CREB
β-actin
E
WCL
Organelle
Cytoplasm
Forskolin - + - + - +
PDE4D
Lamin A/C
Lamp2
α-tubulin

Figure 3

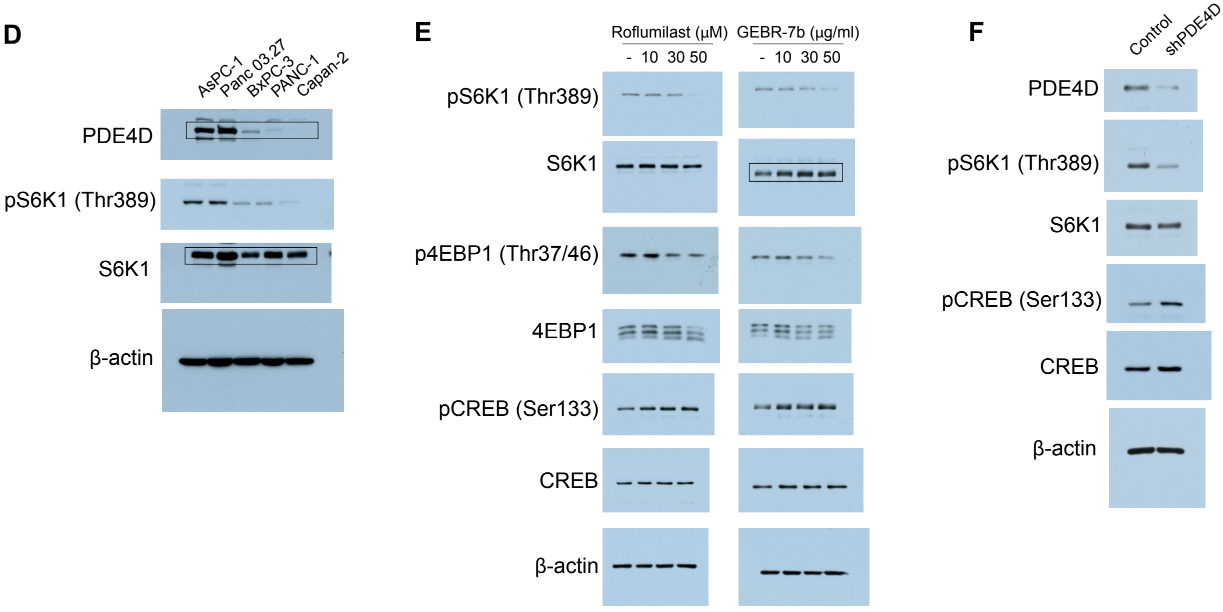
D
AsPC-1
Panc 03.27
BxPC-3
PANC-1
Capan-2
PDE4D
pS6K1 (Thr389)
S6K1
β-actin
E
Roflumilast (μM)
GEBR-7b (μg/ml)
- 10 30 50
- 10 30 50
pS6K1 (Thr389)
S6K1
p4EBP1 (Thr37/46)
4EBP1
pCREB (Ser133)
CREB
β-actin
F
Control
shPDE4D
PDE4D
pS6K1 (Thr389)
S6K1
pCREB (Ser133)
CREB
β-actin

Figure 4

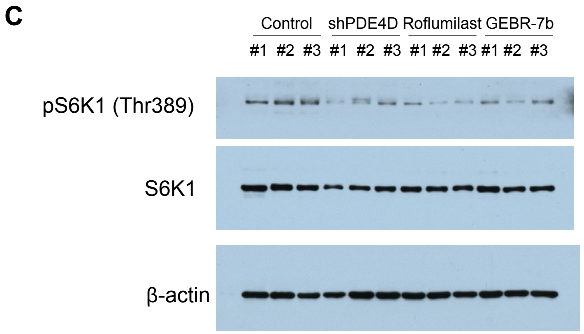
C
Control
shPDE4D
Roflumilast
GEBR-7b
#1 #2 #3 #1 #2 #3 #1 #2 #3 #1 #2 #3
pS6K1 (Thr389)
S6K1
β-actin

Figure 5

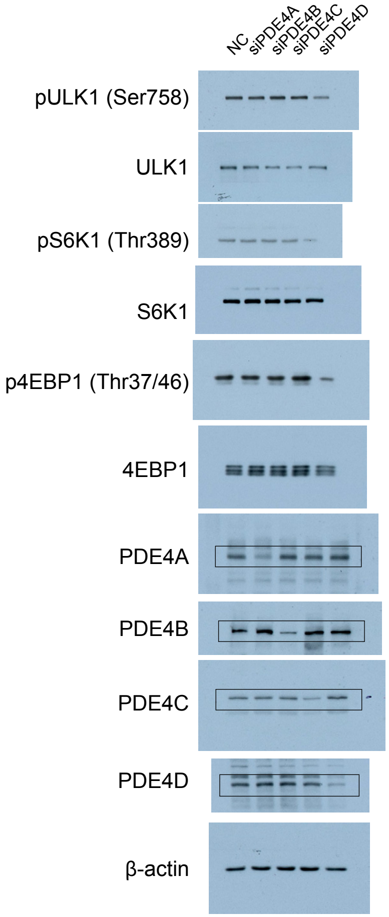

NC
siPDE4A
siPDE4B
siPDE4C
siPDE4D
pULK1 (Ser758)
ULK1
pS6K1 (Thr389)
S6K1
p4EBP1 (Thr37/46)
4EBP1
PDE4A
PDE4B
PDE4C
PDE4D
β-actin

Figure S1

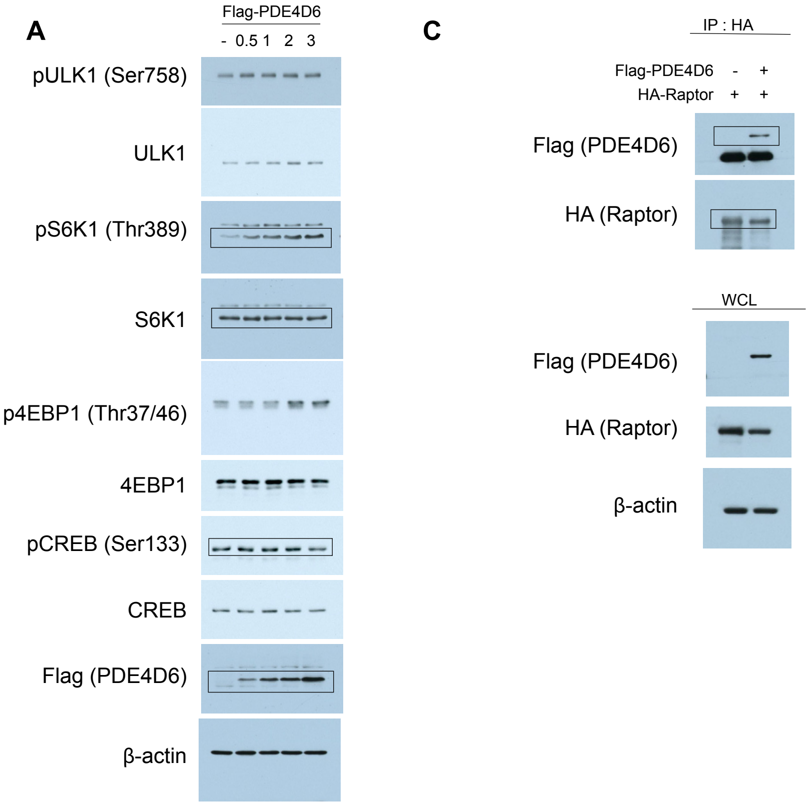
A
Flag-PDE4D6
- 0.5 1 2 3
pULK1 (Ser758)
ULK1
pS6K1 (Thr389)
S6K1
p4EBP1 (Thr37/46)
4EBP1
pCREB (Ser133)
CREB
Flag (PDE4D6)
β-actin
C
IP : HA
Flag-PDE4D6 - +
HA-Raptor + +
Flag (PDE4D6)
HA (Raptor)
WCL
Flag (PDE4D6)
HA (Raptor)
β-actin

Figure S2

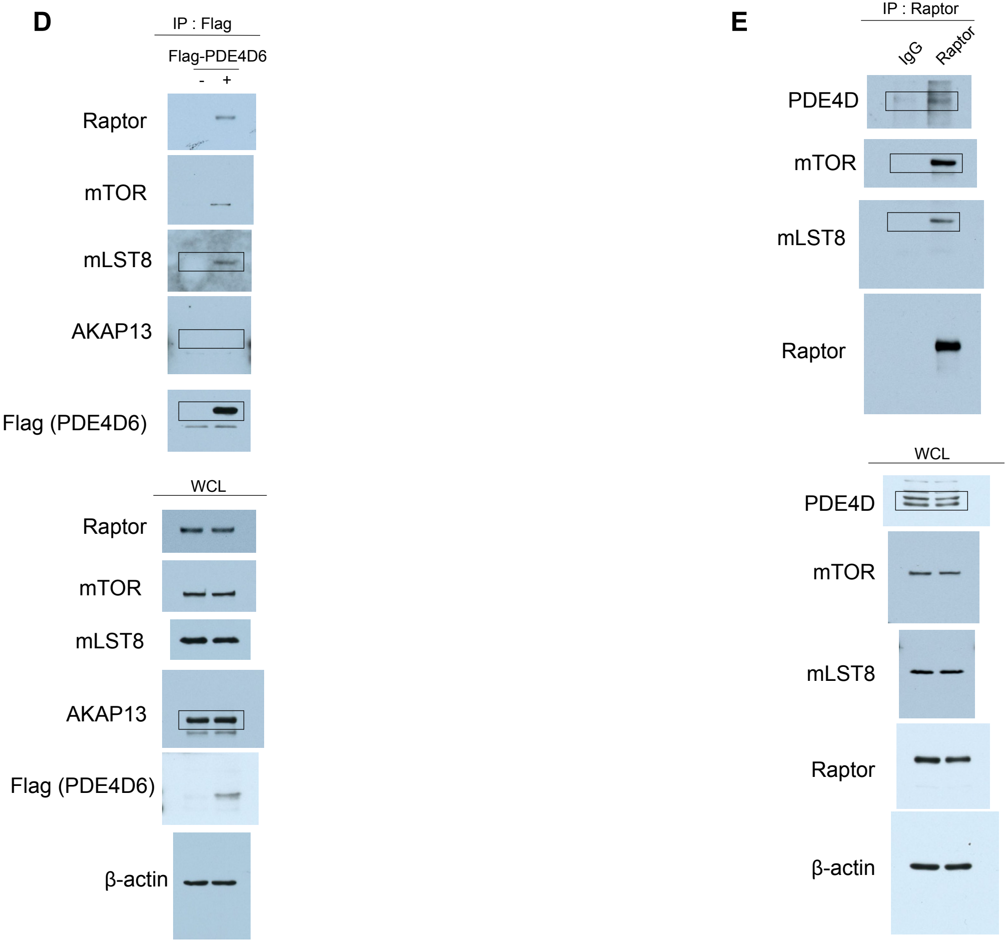
D
IP : Flag
Flag-PDE4D6
- +
Raptor
mTOR
mLST8
AKAP13
Flag (PDE4D6)
WCL
Raptor
mTOR
mLST8
AKAP13
Flag (PDE4D6)
β-actin
E
IP : Raptor
IgG
Raptor
PDE4D
mTOR
mLST8
Raptor
WCL
PDE4D
mTOR
mLST8
Raptor
β-actin

Figure S2

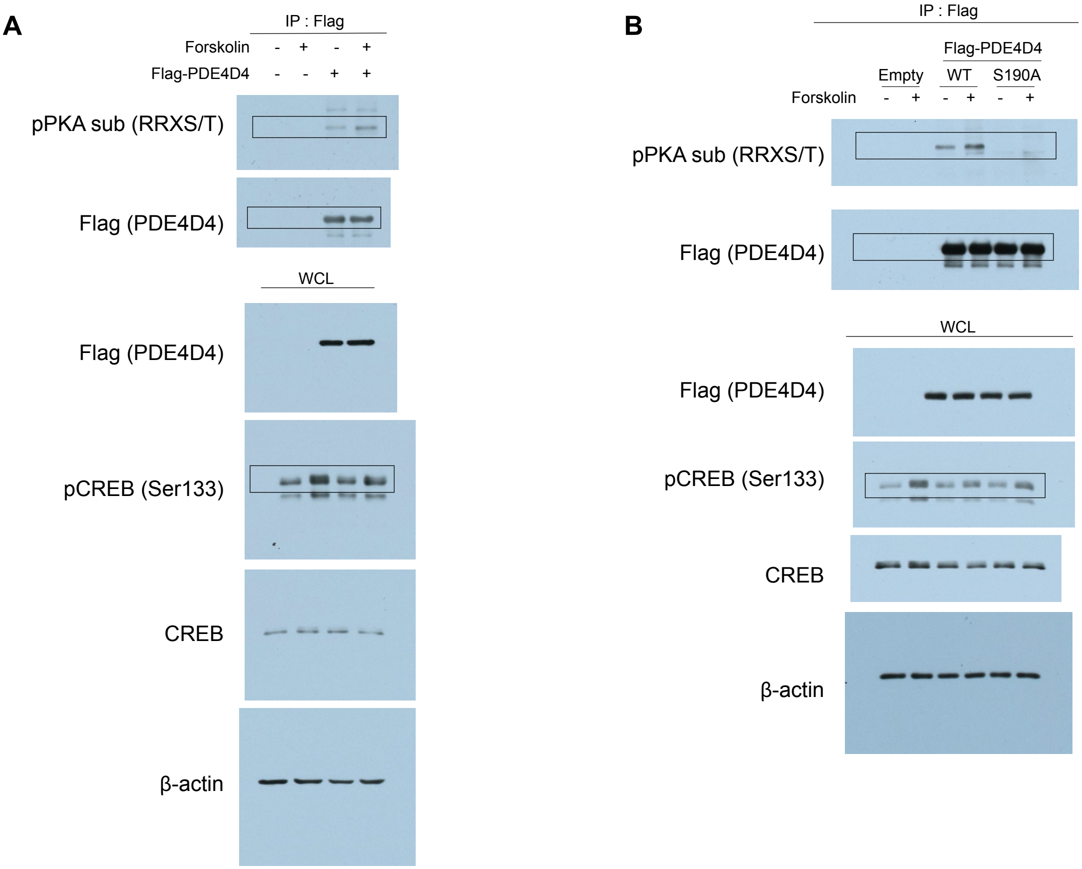
A
IP : Flag
Forskolin - + - +
Flag-PDE4D4 - - + +
pPKA sub (RRXS/T)
Flag (PDE4D4)
WCL
Flag (PDE4D4)
pCREB (Ser133)
CREB
β-actin
B
IP : Flag
Flag-PDE4D4
Empty
WT
S190A
Forskolin - + - + - +
pPKA sub (RRXS/T)
Flag (PDE4D4)
WCL
Flag (PDE4D4)
pCREB (Ser133)
CREB
β-actin

Figure S3

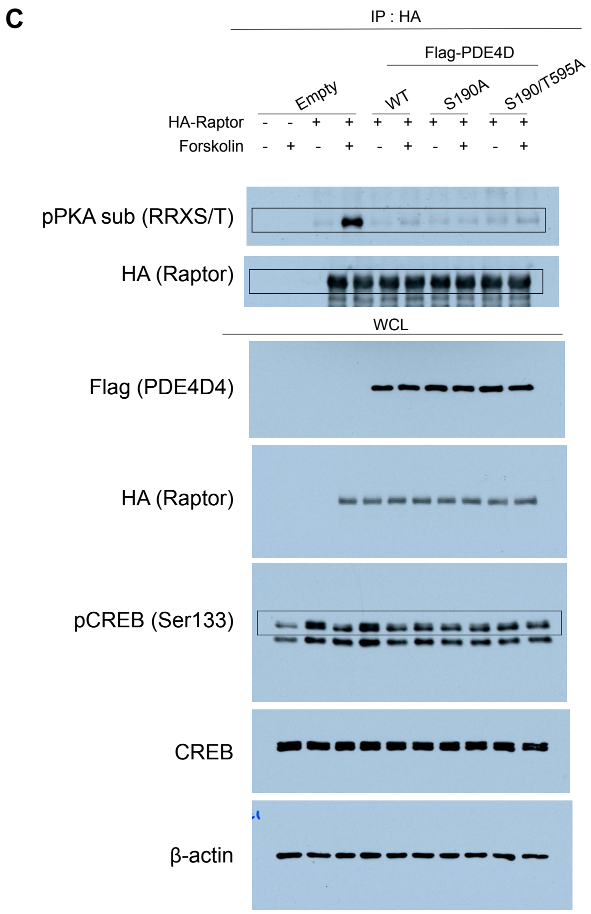
C
IP : HA
Flag-PDE4D
Empty
WT
S190A
S190/T595A
HA-Raptor - - + + + + + + + +
Forskolin - + - + - + - + - +
pPKA sub (RRXS/T)
HA (Raptor)
WCL
Flag (PDE4D4)
HA (Raptor)
pCREB (Ser133)
CREB
β-actin

Figure S3

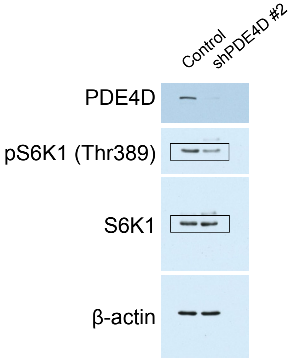

Figure S5

A

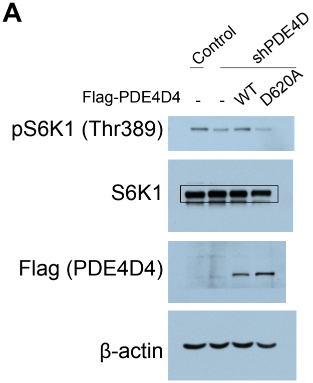
Control
shPDE4D
Flag-PDE4D4
-
-
WT
D620A
pS6K1 (Thr389)
S6K1
Flag (PDE4D4)
β-actin

Figure S6

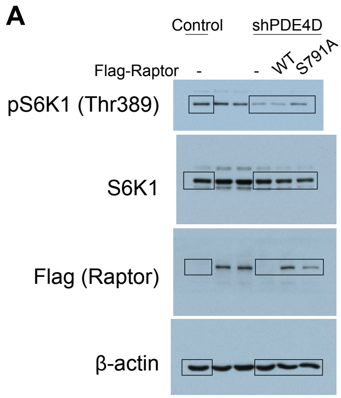
A
Control
shPDE4D
Flag-Raptor
-
-
WT
S791A
pS6K1 (Thr389)
S6K1
Flag (Raptor)
β-actin

Figure S7

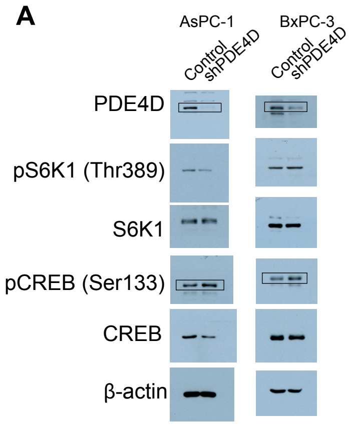
A
AsPC-1
BxPC-3
Control
shPDE4D
Control
shPDE4D
PDE4D
pS6K1 (Thr389)
S6K1
pCREB (Ser133)
CREB
β-actin

Figure S8
